# DNA-binding properties of the MADS-domain transcription factor SEPALLATA3 and mutant variants characterized by SELEX-seq

**DOI:** 10.1007/s11103-020-01108-6

**Published:** 2021-01-24

**Authors:** Sandra Käppel, Ralf Eggeling, Florian Rümpler, Marco Groth, Rainer Melzer, Günter Theißen

**Affiliations:** 1 Matthias Schleiden Institute/Genetics, Friedrich Schiller University Jena, Philosophenweg 12, 07743 Jena, Germany; 2 Department of Computer Science, University of Helsinki, Pietari Kalmin katu 5, 00014 Helsinki, Finland; 3 Methods in Medical Informatics, Department of Computer Science, University of Tübingen, Sand 14, 72076 Tübingen, Germany; 4 Institute for Biomedical Informatics, University of Tübingen, Tübingen, Germany; 5 Leibniz Institute on Aging-Fritz Lipmann Institute (FLI), Core Facility DNA Sequencing, Beutenbergstraße 11, 07745 Jena, Germany; 6 School of Biology and Environmental Science and Earth Institute, University College Dublin, Belfield, Dublin 4, Ireland

**Keywords:** *Arabidopsis thaliana*, A-tract, CArG-box, MADS-box, SELEX-seq

## Abstract

***Key message*:**

We studied the DNA-binding profile of the MADS-domain transcription factor SEPALLATA3 and mutant variants by SELEX-seq. DNA-binding characteristics of SEPALLATA3 mutant proteins lead us to propose a novel DNA-binding mode.

**Abstract:**

MIKC-type MADS-domain proteins, which function as essential transcription factors in plant development, bind as dimers to a 10-base-pair AT-rich motif termed CArG-box. However, this consensus motif cannot fully explain how the abundant family members in flowering plants can bind different target genes in specific ways. The aim of this study was to better understand the DNA-binding specificity of MADS-domain transcription factors. Also, we wanted to understand the role of a highly conserved arginine residue for binding specificity of the MADS-domain transcription factor family. Here, we studied the DNA-binding profile of the floral homeotic MADS-domain protein SEPALLATA3 by performing SELEX followed by high-throughput sequencing (SELEX-seq). We found a diverse set of bound sequences and could estimate the in vitro binding affinities of SEPALLATA3 to a huge number of different sequences. We found evidence for the preference of AT-rich motifs as flanking sequences. Whereas different CArG-boxes can act as SEPALLATA3 binding sites, our findings suggest that the preferred flanking motifs are almost always the same and thus mostly independent of the identity of the central CArG-box motif. Analysis of SEPALLATA3 proteins with a single amino acid substitution at position 3 of the DNA-binding MADS-domain further revealed that the conserved arginine residue, which has been shown to be involved in a shape readout mechanism, is especially important for the recognition of nucleotides at positions 3 and 8 of the CArG-box motif. This leads us to propose a novel DNA-binding mode for SEPALLATA3, which is different from that of other MADS-domain proteins known.

**Supplementary Information:**

The online version of this article (10.1007/s11103-020-01108-6) contains supplementary material, which is available to authorized users.

## Introduction

Widely used large-scale methods to study transcription factor (TF) binding specificity are nowadays ChIP-seq (chromatin immunoprecipitation followed by high-throughput sequencing), protein-binding microarray (PBM) and SELEX-seq (Systematic Evolution of Ligands by Exponential Enrichment followed by high-throughput sequencing) (Orenstein and Shamir 2014; Slattery et al. 2014; Stormo and Zhao 2010). In vivo methods like ChIP-seq enable the study of the biological relevant binding events but are limited in resolution (because fragments are usually quite long) and coverage of all binding sites (especially if only one specific tissue is studied). Additionally, in vivo binding is dependent on several factors, such as chromatin status, binding site accessibility due to nucleosome positioning, binding partners and co-factors (Slattery et al. 2014). In contrast to in vivo binding, in vitro binding is unaffected by these parameters and only direct TF-DNA interactions are detected (Orenstein and Shamir 2014).

The in vitro method SELEX-seq (Smaczniak et al. 2017a) combines the well-established method of SELEX (Ellington and Szostak 1990; Tuerk and Gold 1990) with the power of high-throughput sequencing. SELEX-seq has been shown to be a valuable tool to study transcription factor binding-specificity (Abe et al. 2015; Jolma et al. 2013; Nitta et al. 2015; Sayou et al. 2014; Slattery et al. 2011; Smaczniak et al. 2017b; Wong et al. 2011; Yang et al. 2017; Zykovich et al. 2009). It enables the study of up to 10^7^ or more selected DNA molecules after only one or two selection rounds (Riley et al. 2014; Zykovich et al. 2009). Relative affinities for selected sequences can be obtained by comparing the sequence composition of later rounds to that of the unselected DNA library (Riley et al. 2014).

The specific recognition of DNA target sequences by TFs is essential to control transcription and gene expression. However, the determinants of target gene-specificity are still poorly understood. The elucidation of protein–DNA recognition mechanisms is especially complicated in the case of TF families that use a combination of direct readout (also referred to as “base readout”) and indirect readout (also referred to as “shape readout”) (Rohs et al. 2009, 2010). Direct readout mainly takes place in the DNA major groove by the formation of specific hydrogen bonds between the amino acids of the DNA-binding protein and the DNA bases (Rohs et al. 2010). Indirect readout is a mechanism which describes a variety of interactions between the protein and the DNA whereby the sequence-dependent DNA shape and deformability, such as variations in the minor groove width, the propeller twist or the roll angle of the DNA, are important for recognition (Rohs et al. 2010). Examples for TF that employ both direct and indirect readout are homeodomain proteins (Iyaguchi et al. 2007; Joshi et al. 2007; Li et al. 1998), POU-domain transcription factors (Klemm et al. 1994; Pereira and Kim 2009; Remenyi et al. 2001), histone proteins (Freeman et al. 2014; Rohs et al. 2009; West et al. 2010), HMG proteins (Reeves and Beckerbauer 2001), some nuclear receptors (Gearhart et al. 2003; Meinke and Sigler 1999), several bacterial transcriptional regulators (Alanazi et al. 2013; Fuhrmann et al. 2009; Hong et al. 2005; Shen et al. 2009) as well as MADS-domain transcription factors (Mathelier et al. 2016; Muiño et al. 2014; Rohs et al. 2010).

The MIKC-type MADS-domain protein SEPALLATA3 (SEP3) from the flowering plant model system *Arabidopsis thaliana* is a key regulator of flower development and involved in the determination of floral organs. It acts in a partially redundant manner with the closely related proteins SEP1, SEP2 and SEP4 (Ditta et al. 2004; Kaufmann et al. 2009; Mandel and Yanofsky 1998; Pelaz et al. 2000). SEP3 has recently been shown to use both base and shape readout to achieve binding specificity by both in vivo ChIP-seq experiments, and in vitro gel shift assays and SELEX-seq (Käppel et al. 2018; Mathelier et al. 2016; Muiño et al. 2014; Smaczniak et al. 2017b).

MADS-domain TFs in general bind as dimers to CArG-box sequence elements with the consensus sequence 5′-CC(A/T)_6_GG-3′ or to closely related sequences (e.g. 5′-C(A/T)_8_G-3′) (Folter and Angenent 2006; Melzer et al. 2006; Pellegrini et al. 1995; Schwarz-Sommer et al. 1990; Shore and Sharrocks 1995). X-ray crystal and solution NMR structures of the human MADS-domain TFs SERUM RESPONSE FACTOR (SRF) and MYOCYTE ENHANCER FACTOR 2A (MEF2A) showed that the N-terminal arm of the DNA-binding MADS-domain protrudes deep into the minor groove and that some amino acid residues make contacts with the A/T bases in the center of the CArG-box (Huang et al. 2000; Pellegrini et al. 1995; Santelli and Richmond 2000). The arginine residue at position 3 (R3) of the MADS-domain is part of that N-terminal arm and is highly conserved (Melzer et al. 2006). In a previous site-directed mutagenesis study we have shown that the arginine 3 of SEP3 is important for the recognition of A-tract elements within the CArG-box and for minor groove shape readout (Käppel et al. 2018). However, the investigation had been limited to a set of SRF-type CArG-boxes [consensus 5′-CC(A/T)_6_GG-3′], thus ignoring a possible shift of binding specificity beyond this set that may occur when arginine 3 is mutated into another amino acid.

Here, we employed SELEX-seq to get an extensive overview of the binding repertoire of SEP3. We investigated the importance of the flanking sequences, the preferences for certain sequence patterns within the CArG-box and possible interdependencies between both employing the method SELEX-seq. In order to improve our understanding of the role of the arginine 3 for DNA binding specificity, we substituted it by either a lysine or an alanine residue and studied the binding properties of the mutant proteins.

We demonstrate the preference for a short T-rich sequence 5′ of the CArG-box and for a short A-rich sequence 3′ of the central CArG-box motif. We confirm the importance of A-tract elements within the AT-rich center of the CArG-box for binding affinity. The binding specificity for the AT-rich flanking sequences seems to be widely independent of the central CArG-box motif as suggested by motif complexity analysis (Eggeling 2018). We further demonstrate that the highly conserved arginine 3 residue in the MADS-domain of SEP3 is critical for the recognition of nucleotides at positions 3 and 8 of the CArG-box motif.

## Materials and methods

### Cloning and protein purification

Cloning and protein purification were performed as described previously (Käppel et al. 2018). Here is just a short summary of the strategy.

*SEPALLATA3* (*SEP3*) cDNA (GenBank accession NM_102272, positions 1–270 of the CDS) was cloned into the bacterial expression vector pET-15b (Merck Millipore), thereby creating an N-terminal fused His_6_-tagged protein. The fragment contains DNA encoding the MADS- and the I-domain and was therefore termed *SEP3*_MI_. *SEP3* mutants encoding proteins with single amino acid exchanges (*SEP3*_MI_ R3A and *SEP3*_MI_ R3K) were created by site-directed mutagenesis from the previously created vector carrying *SEP3*_MI_.

Protein purification was done with the help of an Äkta purifier 10. First, the His_6_-tagged proteins were purified on a Ni sepharose column (His-Trap FF crude, GE Healthcare). Bacterial DNA was removed by employing a heparin sepharose column (HiTrap Heparin HP, GE Healthcare). Then the His_6_-tag was removed by thrombin cleavage. Proteins were finally purified using size exclusion chromatography (Superdex75 10/300 GL column, GE Healthcare).

### DNA probe design and preparation

The SELEX-seq experiment was performed essentially as described previously (Riley et al. 2014).

DNA probes were designed to include a central random region of 25 bp, a barcode of 4 bp and flanking regions which are compatible with Illumina sequencing (TruSeq adapter sequences). Probe design is depicted in Supplementary Fig. S1a and probe sequences are listed in Supplementary Table S1.

6 Probes (i.e. 6 SELEX libraries) were prepared for later study of the binding specificity of wildtype SEP3_MI_, and mutant SEP3_MI_ R3A and SEP3_MI_ R3K: probes 1 and 4 were later co-incubated with SEP3_MI_, probes 2 and 5 with SEP3_MI_ R3A and probes 3 and 6 with SEP3_MI_ R3K. Additionally, a positive and a negative control probe were designed. For the positive control the sequence of the CArG box 1 of the *AGAMOUS* intron 2 was used (first described by Hong et al. 2003, shown to be bound by SEP3 in vitro by Kaufmann et al. 2005a). For the negative control the same sequence as for the positive control was used except for the fact that the CArG-box was mutated (Supplementary Table S1). Single-stranded DNA oligonucleotides were purchased from IDT (Integrated DNA Technologies) with the purity degree “standard desalting”. Nucleotides for the 25 random positions were “hand-mixed” (each nucleotide with 25%) to have a higher probability of equal occurrence of each nucleotide. Full-length oligonucleotides (99 nucleotides) representing the forward strand were annealed with the oligonucleotide “Primer_SELEX_rev” (23 nucleotides, Supplementary Fig. S1b; Table S2).

Second strand synthesis was done with Klenow Fragment (Thermo Fisher Scientific). The fill-in reactions were purified using the Gene Jet PCR Purification Kit (Thermo Fisher Scientific).

### Electrophoretic mobility shift assay

The protein–DNA binding buffer was composed as previously described (Käppel et al. 2018). Protein (dimer) concentrations of 0.1 µM were used. Concentration of the DNA probe was 0.2 µM. The binding reaction mixture (20 µl) was incubated for 2 h at 22 °C. Samples were then loaded onto 0.5 × TBE native 5% polyacrylamide gels which had been prepared with a 40% acrylamide/bis-acrylamide solution 19:1. Gels additionally contained 2.5% glycerol for increased gel stability. Gels were pre-run for about 15 min and then run at room temperature at 7.5 V/cm for 3 h. After gel run DNA was stained with ethidium bromide.

### Isolation and elution of bound DNA

The bound DNA of the protein–DNA complex (the shifted band in the gel) was eluted from the gel and purified. After ethidium bromide staining the gel was placed on a transilluminator UV table (Appligene). The band was excised according to the apparent size of the shifted DNA band (protein-bound DNA) of the positive control. The DNA ladder (Thermo Scientific GeneRuler 50 bp DNA Ladder) was also used for orientation since the free DNA probe (99 bp) was visible as a band of about 100 bp, whereas the protein-bound DNA was visible as a band shifted to an apparent size of 150–200 bp compared to the DNA ladder (Supplementary Fig. S2).

The elution of bound DNA was done as previously described (Riley et al. 2014). DNA was stored at − 20 °C.

### DNA amplification and preparation of sequencing libraries

The eluted DNA was amplified via PCR (Supplementary Fig. S1b). 15 to 20 parallel 50 µl PCR reactions were set up per sample, each with the following composition: 5 µl 10 × Dream Taq Buffer, 1 µl 10 mM dNTPs, 2 µl 10 µM Primer_SELEX_fwd, 2 µl 10 µM Primer_SELEX_rev, 0.25 µl (1.25 units) Dream Taq (Thermo Fisher Scientific), 10 µl 5 M betaine (Sigma-Aldrich), 0.5 µl diluted DNA (ca. 0.1 ng) and 29.25 µl nuclease-free water. The thermal cycling program was as follows: initial denaturation at 95 °C for 3 min, then 15 cycles of denaturation at 95 °C for 30 s, annealing at 58 °C for 30 s and extension at 72 °C for 30 s, final extension at 72 °C for 1 min and a hold at 4 °C.

After PCR, reactions were pooled and purified with the Gene Jet PCR Purification Kit (Thermo Fisher Scientific) and then stored at − 20 °C.

For the generation of the sequencing libraries a portion of the initial SELEX libraries as well as of the amplified DNA from the selection rounds 1, 2, 3 and 4 was used. Limited cycle PCR (Supplementary Fig. S1c) was used to add the final adapters to the 30 libraries (6 initial SELEX libraries and the 6 SELEX libraries à 4 selection rounds). The PCR was set up in 5 parallel 50 µl PCR reactions: 0.8 µl 0.5 µM DNA, 25 µl PCR master mix (NEB Next Q5 Hot Start HiFi PCR Master Mix), 4 µl 10 µM Primer_adapter_fwd, 4 µl 10 µM Primer_adapter_rev (dependent on cycle), 16.2 µl nuclease-free water. The thermal cycling program was as follows: initial denaturation at 98 °C for 30 s, then 2 cycles of denaturation at 98 °C for 10 s and annealing/extension at 65 °C for 45 s, followed by 3 cycles of denaturation at 98 °C for 10 s and annealing/extension at 68 °C for 45 s, final extension at 68 °C for 5 min and a hold at 8 °C. After PCR, reactions were pooled and purified with the Gene Jet PCR Purification Kit (Thermo Fisher Scientific). Samples were eluted with 30 µl elution buffer.

The PCR products were then gel-purified. For that purpose they were run on a 0.5 × TBE 5% polyacrylamide gel. After ethidium bromide staining the gel was placed on a transilluminator UV table (Appligene). DNA ladders (Thermo Scientific GeneRuler 50 bp DNA Ladder and Thermo Scientific O’RangeRuler 20 bp DNA Ladder) were used for size orientation of PCR products. The band which ran at an apparent height of 150 bp (PCR product length with complete adapter sequences) was excised (Supplementary Fig. S3). The elution of DNA was done as previously described (Riley et al. 2014).

### Sequencing

Before high-throughput sequencing libraries (Supplementary Fig. S1d; Table S3) were cloned and five clones per library were sequenced by Sanger sequencing. Libraries of the amplification rounds 3 and 4 were mostly found to be highly contaminated with the positive control. This problem could be identified via the specific barcode of the positive control. Highly contaminated samples were excluded from further analysis. Therefore, 20 SELEX libraries were finally sequenced using an Illumina HiSeq2500 running in 51 cycle, single-end, high-output mode. Extraction of read data in FastQ format was done using bcl2fastq v.1.8.4 and resulted after de-multiplexing (for the i7 index: 6mer) in 47,352,383 reads for the initial libraries (R0), 61,282,700 reads for amplification round 1 (R1), 39,737,545 reads for amplification round 2 (R2) and 20,536,537 reads for amplification round 3 (R3). Supplementary Table S4 gives an overview of the sequencing reads for each SELEX library and of the unwanted reads that were obtained for the positive control after de-multiplexing (using the probe index: 4mer).

### Sequencing data analysis

Data analysis of the SELEX-seq experiment was carried out using the SELEX R package (Rastogi et al. 2015) and followed the computational analysis pipeline that was reported previously (Riley et al. 2014). The pipeline contains (i) estimation of an optimal Markov order for the background model, (ii) estimation of an optimal motif length, and (iii) affinity correction.

All k-mers with affinity score > 0.4 were extracted and extended upstream and downstream by a poly-N of length k/2 each. The cutoff was chosen based on the distribution of affinity scores with the aim of obtaining a reasonable tradeoff between maximal information content of the resulting sequence motif and number of the sequences taken into account (50–200, depending on the experiment). The affinities themselves were normalized and upscaled by a factor of 10^3^. Afterwards, de novo motif discovery was carried out using InMoDe (Eggeling et al. 2017) in FlexibleMoDe, searching on both strands for a single position weight matrix (PWM) motif of length 10 by using 10^4^ restarts with different initializations and default values for the remaining external parameters.

For analyzing the flanking sequences of the SRF-type CArG box, all sequences of length 18 matching N_4_CCW_6_GGN_4_ were extracted from the raw reads (of length 25) and strand-oriented by using InMoDe (Eggeling et al. 2017) in FlexibleMoDe with one PWM motif, the length set of which was set identical to sequence length. Motif complexity analysis (Eggeling 2018) was carried out for comparing proximal and distal dependence models of first and second order.

## Results and discussion

### Rationale of the SELEX-seq study

In order to study the DNA-binding properties of the floral homeotic protein SEPALLATA3, we developed a protocol to produce a short version of SEP3 in *Escherichia coli* and to isolate a soluble and functional protein with high purity (Käppel et al. 2018). We obtained an N-terminal fragment of SEP3, which consists of the first 90 amino acids of the 251-amino-acid long full-length SEP3 protein. We termed this truncated protein SEP3_MI_ since it contains the DNA-binding MADS-domain and the I-domain. We have previously shown that SEP3_MI_ is capable of sequence-specific DNA-binding (Käppel et al. 2018). The SEP3_MI_ protein lacks the K- and the C-domain and contains two additional amino acids N-terminal to the MADS-domain. Hence, we cannot completely exclude the possibility that these structural deviations from the native SEP3 protein have an influence on binding specificity. We consider this unlikely, however, since it is well-established that the DNA-binding specificity of MADS-domain proteins is largely determined by the sequence of the MADS-domain proper (Kaufmann et al. 2005b).

Also, two mutant versions of this protein were studied. For SEP3_MI_ R3A and SEP3_MI_ R3K, which have a single amino acid substitution at the third position of the MADS-domain, we have already shown that they have an altered DNA-binding specificity (Käppel et al. 2018). However, how the binding motif differs between these mutant proteins and the wildtype protein has not been determined yet.

**Fig. 1:**
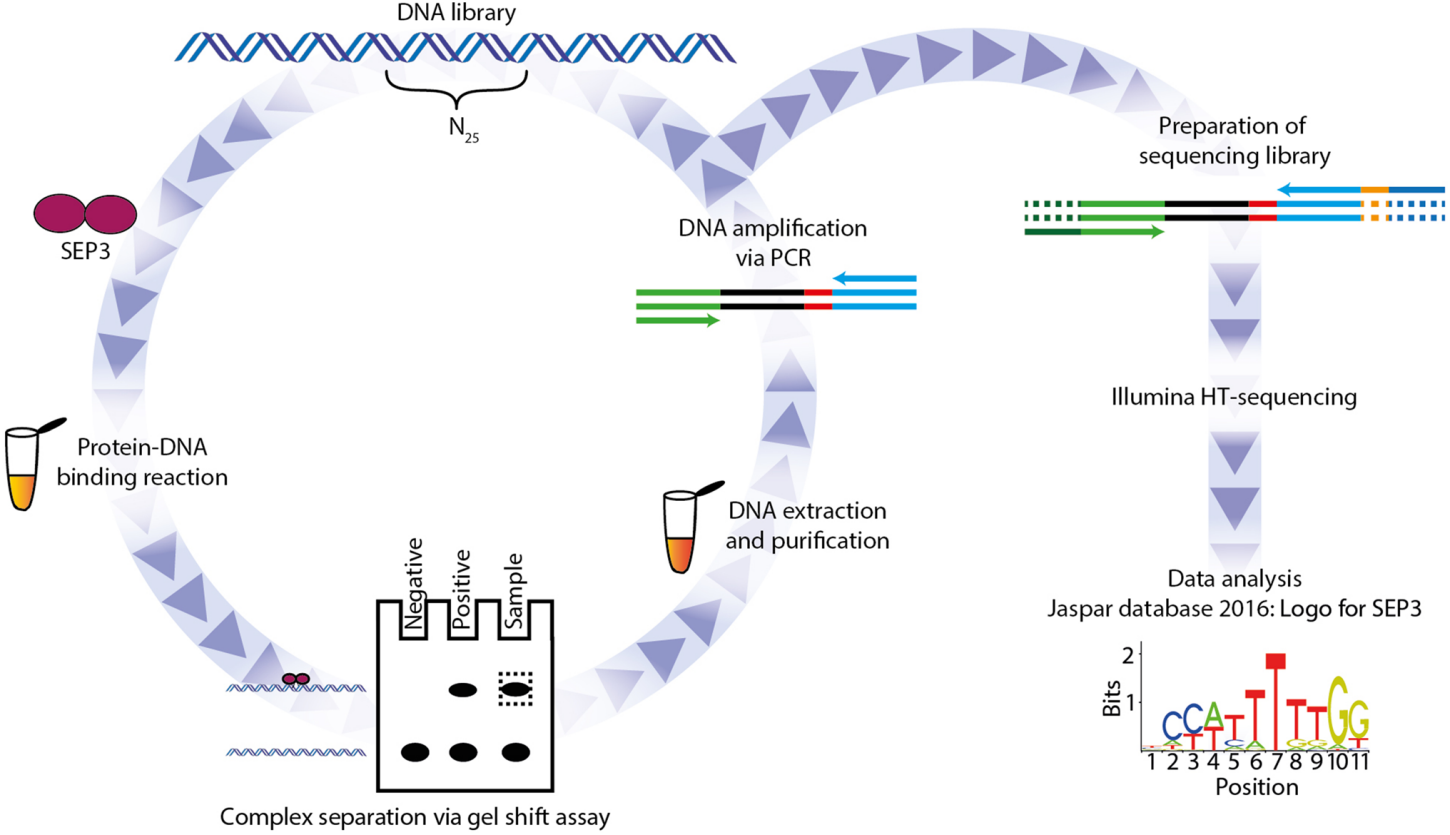
Random DNA libraries with a length of 25 base pairs, which were flanked by defined adapter sequences for PCR amplification and sequencing, were used as a starting point. These DNA libraries were co-incubated with the protein of interest (here: SEP3_MI_, SEP3_MI_ R3A or SEP3_MI_ R3K protein dimers, respectively) in protein–DNA binding reactions. Then, protein-bound DNA and free DNA were separated by native polyacrylamide gel electrophoresis in gel shift assays. The fraction of protein-bound DNA was extracted from the gel, purified and amplified via PCR. One part of this amplified DNA was used for the next round of selection and the other part was prepared for high-throughput sequencing. Sequencing libraries were prepared by the addition of final adapters via limited cycle PCR and subsequent gel purification of PCR products. SELEX libraries were sequenced with an Illumina HiSeq. Data was analyzed using a previously described pipeline (Riley et al. 2014) as well as by newly developed techniques

SELEX-seq (Systematic Evolution of ligands by exponential enrichment followed by high-throughput sequencing) is a method that enables the study of protein–DNA interactions without prior knowledge of the DNA-binding specificity of the protein of interest. A general overview of the SELEX-seq protocol is given in Fig. 1. In order to create DNA libraries that represent the binding repertoires of SEP3_MI_ and the mutant proteins, four rounds of SELEX selection were conducted with probes containing random 25mers in the center (Fig. 1). DNA target sequences of SEP3_MI_ were isolated via a gel shift assay (Supplementary Fig. S2). The fraction of DNA which was bound by the protein was purified from the gel and used for the next amplification round. Due to technical reasons (see “Materials and methods” section) SELEX round R2 was chosen to analyze the binding properties of SEP3_MI_ and its mutated versions.

After Illumina sequencing of the DNA libraries we followed the computational pipeline described by Riley et al. (2014). The first step involved the modeling of biases in the initial libraries, which may arise from different sources like biased DNA synthesis, biases in PCR amplification, etc. The optimal order for the Markov model that best captures the biases in the initial R0 random DNA libraries was determined for each data set (Supplementary Fig. S4). In the second step, the effective length of the DNA-binding site was determined. The optimal motif was estimated to have a length of 10 base pairs, because the information gain was maximal for a Kmer length of 10 (Supplementary Fig. S5).

In the third step, relative affinities were calculated by determining the relative enrichment of motifs from round R0 (initial random DNA libraries) to SELEX round R1 and round R2 (Supplementary Fig. S6). Refinement of affinity estimates was done by LOESS regression (Supplementary Fig. S7). The purpose is to obtain an appropriate tradeoff between low bias and low variance of the relative affinity estimate.

### SEP3_MI_ shows the typical preference for CArG-boxes with an A-tract after binding site enrichment by SELEX

**Fig. 2:**
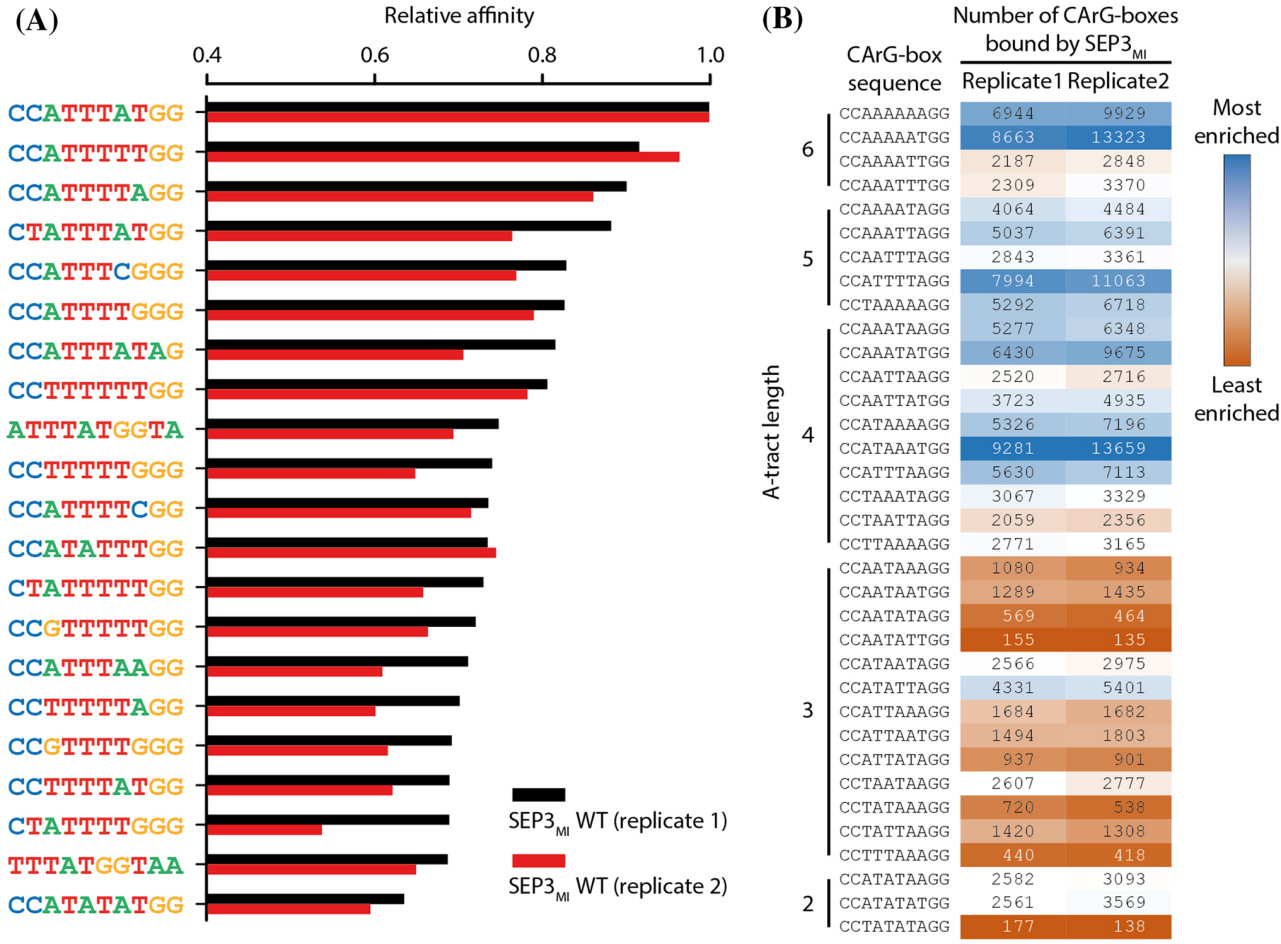
High-affinity binding sequences of SEP3_MI_. **a** In this bar chart the relative affinities of the 21 10mers are shown which had the highest affinities in both replicates of the SELEX experiment with SEP3_MI_. SEP3_MI_ shows a binding preference for CArG-boxes and CArG-box-like sequences. **b** Heat map showing the importance of A-tract length within SRF-type CArG-boxes for the DNA-binding ability of SEP3_MI_. The counts comprise occurrences of both the shown sequence and its reverse complement. CArG-box sequences with A-tracts (A_n_T_m_, n + m ≥ 4) are enriched among the SEP3_MI_ binding sites compared to CArG-box sequences without a true A-tract (A_n_T_m_, n + m ≤ 3). The most enriched CArG-box sequences contain an A-tract (blue color), whereas the least enriched CArG-box sequences do not (red color)

Both replicates of the SELEX experiment with the SEP3_MI_ protein show a clear binding preference of SEP3_MI_ to the SRF-type CArG-box motif 5′-CC(A/T)_6_GG-3′ (Fig. 2a). Among the 21 sequences with the highest relative binding affinities of each replicate are 9 SRF-type CArG-boxes. The other sequences with high binding affinity mostly include sequences with an AT-rich center of 4 to 7 nucleotides and C/G borders of 1 to 4 nucleotides flanking the AT-rich center.

Some of the bound sequences seem to contain incomplete CArG-boxes, such as the sequence 5′-TTTATGGTAA-3′, where the CC or GG borders, and sometimes also parts of the AT-rich CArG-box center are missing. We investigated the localization of these 10mer binding motifs within the random 25mer sequences of the DNA probes and found a strong position bias towards the 5′-end of the probe (Supplementary Fig. S8), in particular an enrichment at positions 1 and 2. Since the 3′-end of the Illumina sequencing adapter is a CT dinucleotide (see Supplementary Table S1 for probe sequences), a plausible explanation is that some protein binding occurred in the constant 5′-part of the DNA probes.

In order to learn a single position weight matrix model from complete and incomplete CArG-boxes, we used the following procedure. We considered all 10mers with an affinity score greater than 0.4 and extended them upstream and downstream with five ambiguous nucleotides (N in IUPAC code)_._ Subsequently, we performed a de novo motif discovery using a motif length of 10 (see “Materials and methods” section for details), so that complete and incomplete CArG-boxes become aligned.

**Fig. 3:**
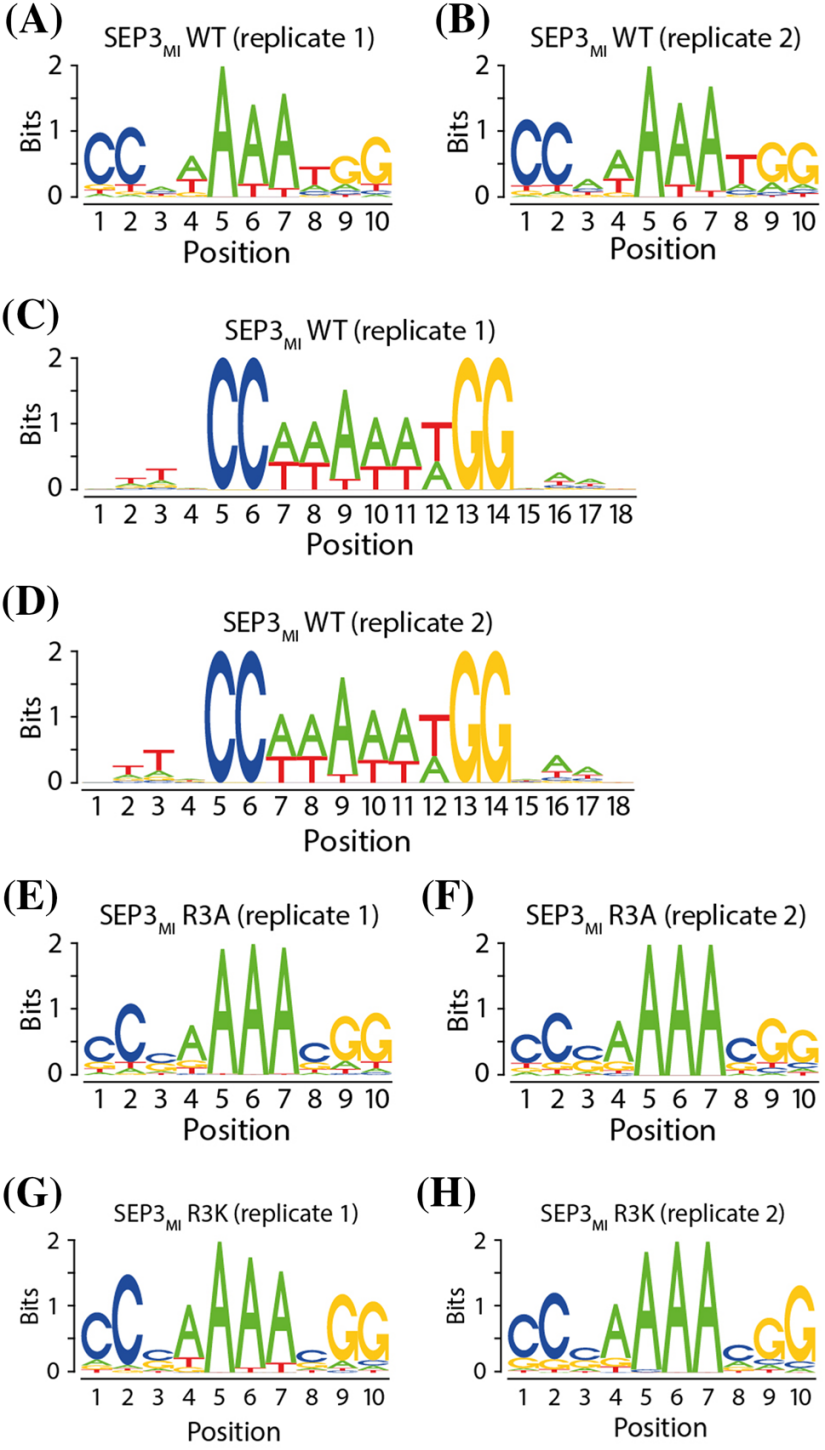
Sequence logos of SEP3_MI_. **a** and **b** Sequence logos were generated by de novo motif discovery whereby the motif length was set to 10, affinity values were normalized and only sequences with a relative affinity ≥ 0.4 were considered. The sequence logo of the wildtype (WT) SEP3_MI_ protein depicts a typical CArG-box sequence of the type CC(A/T)_6_GG. The preferential central sequence is 5′-NWAAAT-3′. Both experimental replicates produced very similar results. **c** and **d** Sequence logos of the flanking sequences of SRF-type CArG-boxes obtained by a *guided* motif discovery. 4 Base pairs upstream and 4 base pairs downstream, respectively, of every occurrence of a perfect SRF-type CArG-box of the form 5′-CC(A/T)_6_GG-3′ were investigated. The preferred flanking sequences are a very short T-rich sequence at the 5′-end (5′-TTN) and a very short A-rich sequence at the 3′-end (NAA-3′). Both experimental replicates produced very similar results. **e**–**h** Sequence logos of the mutant proteins SEP3_MI_ R3A and SEP3_MI_ R3K. Sequence logos were generated as described for **a** and **b**

The resulting sequence logo of SEP3_MI_ (Fig. 3a, b) shows a preference for a CArG-box center as follows: 5′-NWAAAT-3′. This is reminiscent of A-tract sequences which can be defined as sequences which contain at least four consecutive A·T base pairs without an intervening TpA step (i.e. A_n_T_m_, n + m ≥ 4) (Hud and Plavec 2003; Stefl et al. 2004).

We had a closer look at the binding affinities of the 36 SRF-type CArG-boxes with the consensus sequence 5′-CC(A/T)_6_GG-3′. We wanted to see whether we would find systematic differences in DNA-binding affinity between CArG-box sequences with or without an A-tract. We found that CArG-boxes with an A-tract (A_n_T_m_, n + m ≥ 4) were bound more frequently than CArG-boxes without an A-tract (A_n_T_m_, n + m ≤ 3), e.g. the 10 most enriched CArG-box sequences contain an A-tract, whereas the 10 least enriched CArG-box sequences do not (Fig. 2b). This confirms previous data on the binding preference of SEP3 for A-tract containing CArG-box sequences (Käppel et al. 2018; Muiño et al. 2014).

### SEP3_MI_ shows a preference for certain motifs flanking the central CArG-box motif

In addition, we studied the flanking sequences, i.e. four base pairs upstream and four base pairs downstream, respectively, of the SRF-type CArG-box binding motif 5′-CC(A/T)_6_GG-3′ (see “Materials and methods” section for details). We found a preference for a very short T-rich sequence 5′ of the CArG-box and for a very short A-rich sequence 3′ of the central CArG-box motif (Fig. 3c, d). These observations coincide with previous analyses of ChIP-seq data of eight MADS-domain proteins in *A. thaliana* in which also an “NAA extension” of the CArG-box motif was identified (Aerts et al. 2018). Similarly, some other authors reported that the prevalent flanking sequences in their SELEX-seq study, which they conducted with five different MADS-domain TF complexes (including also SEP3 homodimers), are the sequence motifs 5′-TTN- and -NAA-3′ (Smaczniak et al. 2017b).

### Weak dependencies between flanking sequences and motif core

In order to study possible dependencies between the “NAA extensions” and the center of the CArG-box motif, we carried out a recently proposed method called motif complexity analysis (Eggeling 2018), which allows to learn different models of intra-motif dependency and compare them using a complexity measure based on information theory. We compared proximal dependence models, which allow dependencies only among adjacent nucleotides, with distal dependence models, which have no such restrictions. For both model types, we considered models of first and second order (order: number of sequence positions a single nucleotide is allowed to depend upon).

We then compared the intra-motif complexity (IMC) measures of our models (Table 1). The minimal value for such a study is zero, which would mean that no dependency exists. The IMC measures of our models are relatively low with values between 1.5 and 2.4. For other TF, IMC values up to 20 have been observed (Eggeling 2018), which means that strong dependencies were detected. There are only small differences in the conditional probabilities for each position in the binding motif (Supplementary Fig. S9).

**Table 1:** Intra-motif complexity (IMC) measures of different types of motif models

Model	Order	SEP3_MI_ WT (Replicate 1)	SEP3_MI_ WT (Replicate 2)
Proximal	1	1.471	1.665
Proximal	2	1.752	2.010
Distal	1	1.633	1.901
Distal	2	2.004	2.398

The values quantify the amount of statistical dependencies in the data that can be effectively represented by a particular motif representation. Here, proximal and distal dependence models of first and second order are compared. Since a PWM model has an IMC value of 0 by definition, the results allow to conclude that most additional information beyond mononucleotide frequencies is given by correlations among directly neighboring nucleotides (first-order proximal dependencies). Increasing the model flexibility, either by increasing the order or by allowing distal dependencies yields only a small further improvement in comparison

Moreover, most of the intra-motif complexity is already covered by a model that only takes into account first-order dependencies among directly adjacent nucleotides. Even when extending the model complexity, larger percentage gains are achieved by increasing the model order rather than by changing the model type.

These observations suggest the absence of strong intra-motif dependencies between the central CArG-box motif and its neighboring sequence fragments in contrast to what has been described for binding motifs of other transcription factor families (Eggeling et al. 2015; Keilwagen and Grau 2015; Nitta et al. 2015; Slattery et al. 2011).

### A conserved arginine residue is important for the recognition of nucleotides at positions 3 and 8 of the CArG-box motif

Recently, we described the phenomenon that the arginine residue 3, which is located in the N-terminal arm of the DNA-binding MADS-domain of SEP3, is important for minor groove shape readout (Käppel et al. 2018). In order to improve the understanding of the role of this arginine residue for DNA binding specificity, the arginine residue 3 in SEP3_MI_ was substituted by either a lysine (which is a basic amino acid like arginine) or an alanine residue. The binding properties of the mutant proteins SEP3_MI_ R3K or SEP3_MI_ R3A, respectively, were then compared to the wildtype protein SEP3_MI_.

**Fig. 4:**
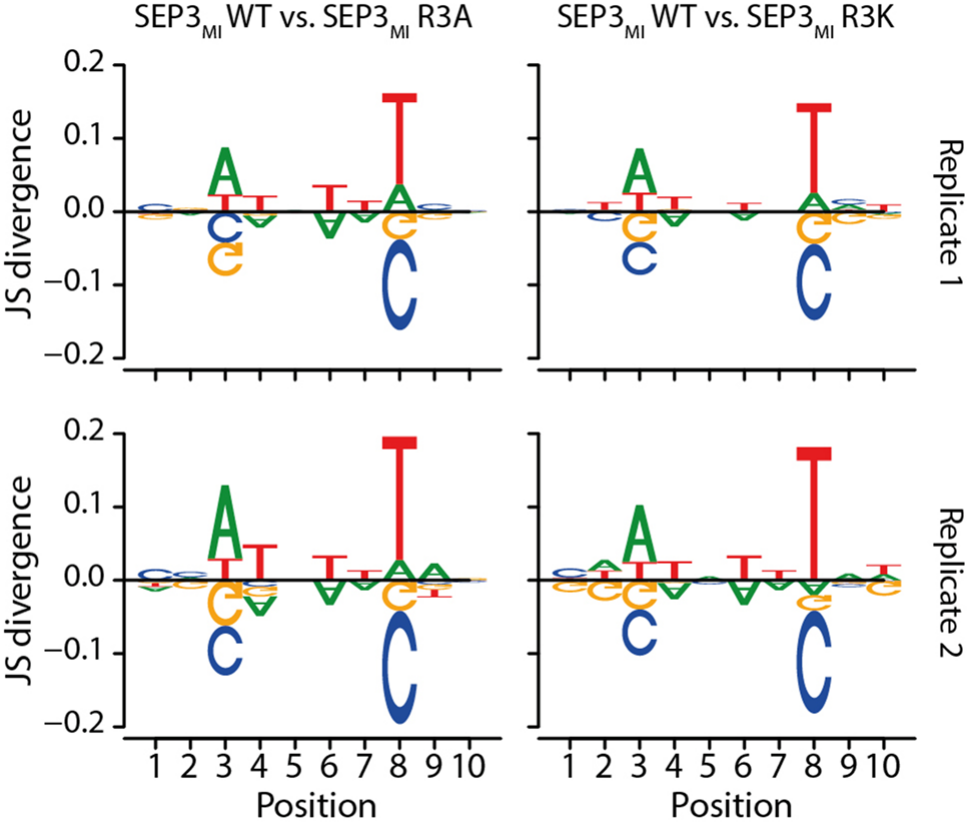
Difference logos created with *DiffLogo* (Nettling et al. 2015). The DNA-binding motif of the wildtype protein SEP3_MI_ is compared to the sequence motifs of the mutant proteins SEP3_MI_ R3A and SEP3_MI_ R3K. Replicates 1 (upper panels) and replicates 2 (lower panels) are being analyzed

To this end, we carried out the same computational analysis as for SEP3_MI_. The sequence logos of SEP3_MI_ R3A and SEP3_MI_ R3K, that combine partial and complete CArG-boxes of the top oligonucleotides according to affinity score are shown in Fig. 3e–h. For easier visual comparison between replicates and between protein variants, we also created difference logos using *DiffLogo* (Nettling et al. 2015) (see Fig. 4; Supplementary Fig. S10). Whereas no big differences can be seen between either replicates (Supplementary Fig. S10a–c, quantified in Table 2) or between the two mutant proteins (Supplementary Fig. S10d, e; Table 2), there are several differences between the binding motifs of the mutant proteins compared to the binding motif of the wildtype SEP3_MI_ protein (see Fig. 4, quantified in Table 2).

**Table 2:** Jenson–Shannon divergence among PWMs

SEP3_MI_ protein		R3A (1)	R3K (1)	WT (2)	R3A (2)	R3K (2)
WT	(1)	0.487	0.428	0.032	0.595	0.548
R3A	(1)	–	0.115	0.538	0.088	0.179
R3K	(1)	–	–	0.464	0.157	0.166
WT	(2)	–	–	–	0.658	0.605
R3A	(2)	–	–	–	–	0.140

There are obvious differences between the SEP3_MI_ WT protein versus the mutant proteins SEP3_MI_ R3A and SEP3_MI_ R3K. In these cases the values for the Jenson–Shannon divergence are high. There are no clear differences between either replicates (1)/(2) or between the two mutant proteins, which can be seen from low values for the Jenson–Shannon divergence

While positions 1 and 2 of the 10-base-pair-long motif are in most cases a cytosine and positions 9 and 10 are mostly occupied by a guanine, the 6 central positions of the CArG-box motif (here positions 3–8) show several differences between the binding motifs of the wildtype and the mutant proteins. Most strikingly are the differences at positions 3 and 8 (Fig. 4).

The wildtype protein shows no clear base preference at position 3 (Fig. 3a, b). In contrast, the mutant proteins prefer either cytosine or guanine (Fig. 3e–h). The difference logos show that the sequence preference at position 3 shifts from a weak preference for A/T for the wildtype protein to a C/G preference for the mutant protein variants (Fig. 4).

Position 8 is dominated by thymine in case of the wildtype protein (Figs. 3a, b, 4), whereas there is again a preference for either cytosine or guanine in case of the mutant variants (Figs. 3e–h, 4).

The difference at position 4 is weaker than the previous differences. Still, the wildtype protein preferentially binds an adenine or a thymine and a guanine in some cases (Fig. 3a, b), while the mutant proteins prefer to bind an adenine and with lower frequency a guanine or a thymine at this position (Fig. 3e–h). However, adenine seems to slightly dominate position 4 for all three proteins. As can be seen from the difference logos the wildtype protein binds a thymine with higher frequency at position 4, whereas adenine is bound more frequently by the mutant protein variants (Fig. 4).

Positions 5 to 7 of the binding motif are highly dominated by adenines for both the wildtype and the mutant proteins. Whereas, the wildtype protein also accepts thymines at positions 6 and 7 (Fig. 3a, b), the mutant proteins almost exclusively prefer adenines at these three positions (Fig. 3e–h). The difference logos visualize that the wildtype protein also tolerates thymine at positions 6 and 7, while adenine is strongly preferred by the mutant protein variants (Fig. 4).

In summary, the consensus sequence of the wildtype protein can be depicted as 5′-CCNWAAATGG-3′, whereas the consensus sequence of the mutant proteins is rather 5′-CCSAAAASGG-3′. This means that the A/T-rich CArG-box core comprises 5 to 6 nucleotides in case of the wildtype protein SEP3_MI_ (positions 3–8), however for the mutant proteins SEP3_MI_ R3A and SEP3_MI_ R3K the A/T-rich sequence is shorter with a length of only 4 nucleotides (positions 4–7). We conclude that the arginine residue 3 of the N-terminal extension of the MADS-domain of SEP3 is especially important for the recognition of nucleotides at positions 3 and 8 of the CArG-box motif.

### Comparison of our proposed binding mode of SEP3 to the DNA-binding properties of the MADS-domain proteins MEF2A and SRF

Most likely a gene duplication event followed by gene divergence led to the split-up of MADS-box genes into two lineages in the most recent common ancestor (MRCA) of extant eukaryotes: type I and type II MADS-box genes (Gramzow and Theißen 2010). Whereas type I MADS-box genes probably evolved into the animal SRF lineage and into the plant type I MADS-box genes, which possess an SRF-like MADS-domain, the type II lineage evolved into the MEF2 lineage in animals and MIKC-type MADS-box genes in plants, which possess a MEF2-like MADS-domain (Gramzow and Theißen 2010).

**Fig. 5:**
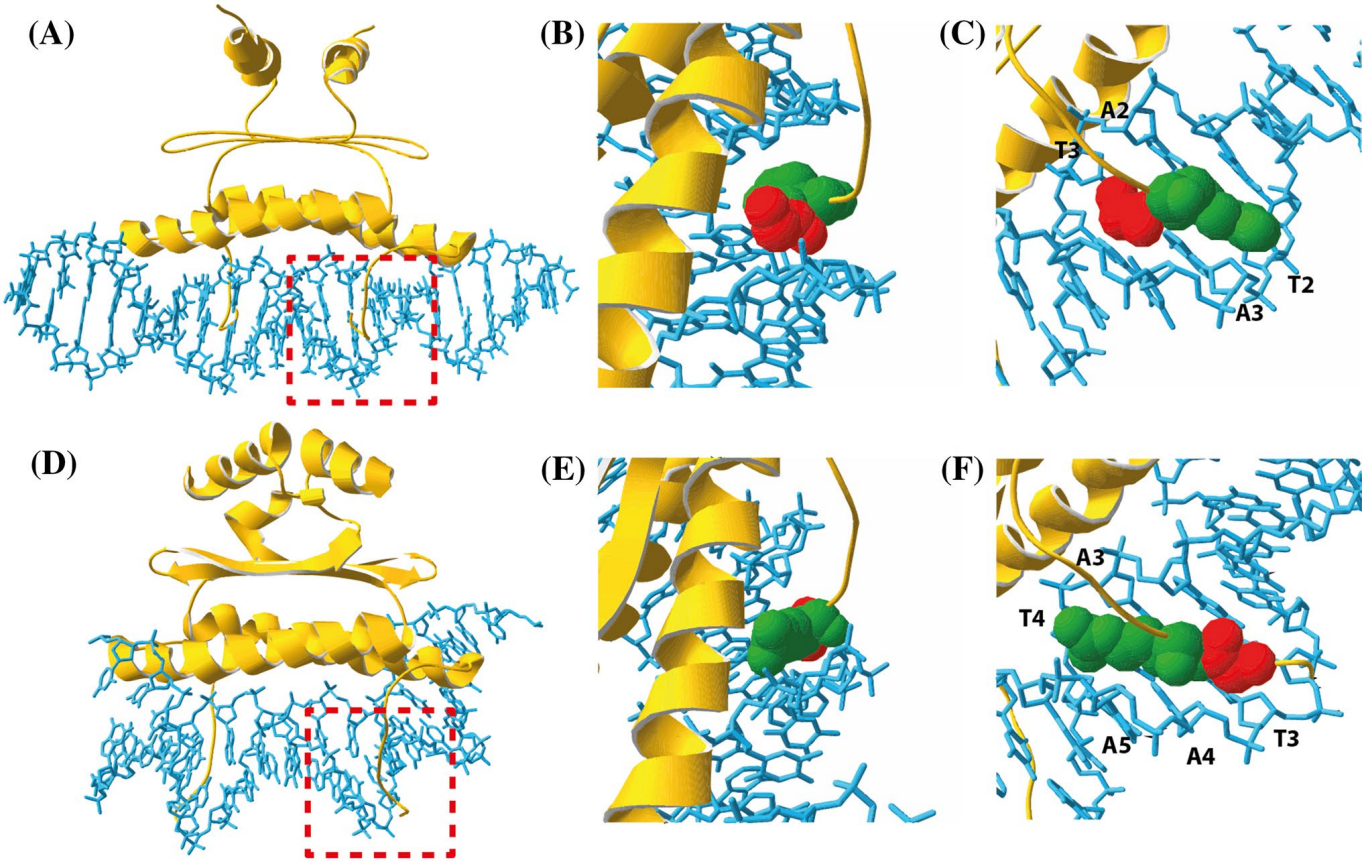
Reversed orientation of glycine 2 and arginine 3 in SRF and MEF2A. **a** Solution structure of the MADS-domain of MEF2A (Protein Data Bank accession 1C7U Huang et al. 2000). The red box highlights the N-terminal arm of MEF2A which is shown in close-up pictures in **b** and **c**. In **b** and **c** glycine 2 and arginine 3 residues, which are buried deep within the DNA minor groove, are shown as red and green space-filling models, respectively. Bases, that are contacted by arginine 3, are labeled. **d**–**f** Crystal structure and corresponding close-up pictures with highlighted glycine 2 and arginine 3 residues of the MADS-domain of SRF (Protein Data Bank accession 1SRS Pellegrini et al. 1995). The figure was created with the Swiss-Pdb Viewer software (Guex and Peitsch 1997)

**Fig. 6:**
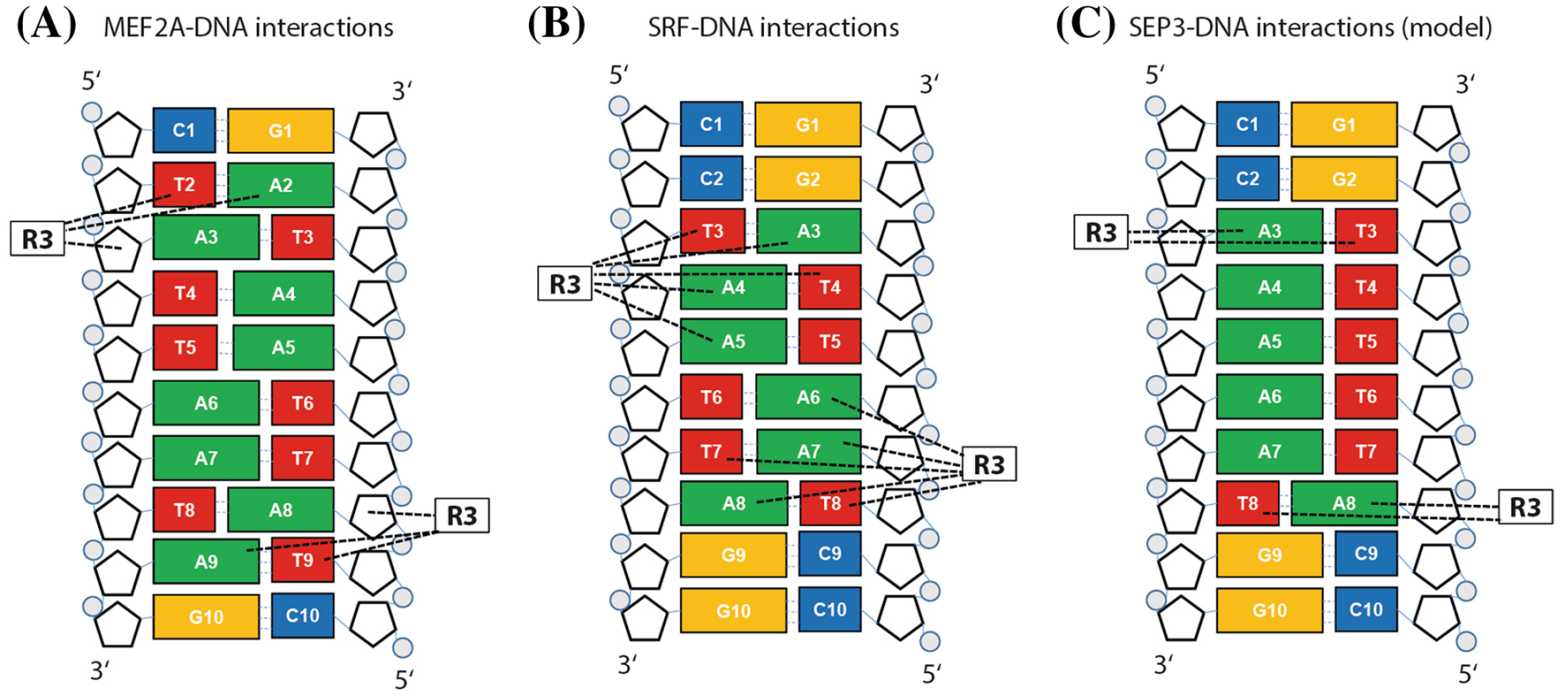
Schematic overview of the DNA contacts of the amino acid arginine 3 of the MADS-domain transcription factors **a** MEF2A, **b** SRF and **c** SEP3. The bases are depicted as rectangles, the deoxyribose sugar is depicted as a pentagon and the phosphate is shown as a grey circle. Both arginine 3 residues (R3) from the protein dimers are shown as rectangles. The black dashed lines indicate the interactions of arginine 3 and the DNA in the minor groove. The numbering scheme of the MEF2A-DNA and the SRF-DNA complex have been converted to that of the proposed SEP3-DNA complex for ease of comparison. **a** The structure of the MEF2A-DNA complex has been published with the pdb identifier 1C7U and was taken from (Huang et al. 2000). **b** The structure of the SRF-DNA complex has been resolved with the respective pdb identifier 1SRS and was taken from (Pellegrini et al. 1995). **c** The SEP3-DNA complex is a hypothetical model that we infer from our DNA-binding data. The base contacts of arginine 3 are unknown. The base contacts of R3, that are shown here, are hypothetical, therefore

There are crystal and solution structures available for the MADS-domain proteins SRF and MEF2A bound to DNA (Huang et al. 2000; Pellegrini et al. 1995; Santelli and Richmond 2000) (Fig. 5). Although both proteins bind as protein dimers to the (almost) palindromic CArG-box sequence via the MADS-domain and though both transcription factors employ the N-terminal extension of the MADS-domain to contact the narrow minor groove of the AT-rich CArG-box center, there are also some differences in the DNA binding properties. While SRF binds to the SRF-type CArG-box with the consensus 5′-CC(A/T)_6_GG-3′ (Pellegrini et al. 1995), MEF2A binds to CArG-boxes with the consensus sequence 5′-C(A/T)_8_G-3′ (Huang et al. 2000). While for both proteins the arginine residue 3 of the N-extension is essential for the DNA contacts, this conserved amino acid makes different base and sugar contacts in the protein–DNA complexes (Figs. 5, 6).

In both SRF and MEF2A the N-terminal arm of the MADS-domain contacts the DNA minor groove. Especially glycine 2 and arginine 3 are buried deep within the minor groove. However, the direction of the polypeptide chain is reversed between SRF and MEF2A (Fig. 5). The side chain of arginine 3 in SRF occupies the same position as the backbone of glycine 2 in MEF2A. Also, the backbone of glycine 2 in SRF is at the same position as the side chain of arginine 3 in MEF2A (Huang et al. 2000).

The consequence of this orientation is that arginine 3 in MEF2A contacts the bases T2 and A2 and the sugar of A3 (the other arginine 3 residue of the dimer contacts bases A9 and T9 and the sugar of A8) (Figs. 5a–c, 6a, the numbering scheme has been changed to that of the proposed SEP3-DNA complex). Glycine 2 of MEF2A contacts the base T3 (Fig. 5c). In SRF arginine 3 contacts the bases T3, A3, A4, T4 and A5 (or A6, T7, A7, A8 and T8, respectively), while the backbone of glycine 2 contacts the sugar of T3 (Figs. 5d–f, 6b).

The direction of the main chain adopted by residues 2 and 3 in MEF2A is impossible in SRF due to the presence of residues N-terminal to the MADS-domain. The conformation of the N-terminal arm observed in MEF2A is excluded in SRF owing to steric clash. In summary, it can be stated that the interactions between glycine 2 and arginine 3 with the DNA are key determinants in binding specificity (Huang et al. 2000).

Our proposed binding mode for SEP3 seems to be neither identical to the DNA-binding properties of SRF nor to those of MEF2A. SEP3 preferentially binds to a CArG-box with the same consensus sequence like SRF, namely the SRF-type CArG-box with the consensus 5′-CC(A/T)_6_GG-3′. However, our suggested base contacts of arginine 3 (R3) of SEP3 to the base pairs at position 3 and 8, respectively, (Fig. 6c) are more similar to MEF2A than to SRF. This is also due to the fact that in SRF 140 amino acids N-terminal to the MADS-box are present—in contrast to MEF2A or SEP3—and thereby a different conformation of the N-terminal extension of SRF in the minor groove of the DNA has to be adopted in comparison to MEF2A and most likely also in comparison to SEP3.

### The role of arginine 3 for DNA-binding specificity of plant MIKC-type MADS-domain TFs

Arginine R3 is highly conserved among plant MIKC-type MADS-domain TFs (Käppel et al. 2018). This strong conservation of the arginine residue in evolution suggests that R3 is not only important for SEP3, but equally important for the DNA-binding properties of other MIKC-type MADS-domain TFs. However, the question remains as to whether this arginine residue is more important for DNA-binding affinity or specificity. We have shown previously that R3 is very important for DNA-binding affinity: compared to the wildtype protein the mutant proteins SEP3_MI_ R3A and SEP3_MI_ R3K showed a reduced DNA-binding affinity to a high-affinity CArG-box by around 4- to 7-fold and to a low affinity CArG-box by around 13- to 14-fold (Käppel et al. 2018). The high conservation of arginine 3 and its importance for DNA-binding affinity seem not to make it a hot candidate for DNA-binding specificity within the MIKC-type MADS-domain TF family. However, there is still a possibility that R3 can help to confer target gene specificity between different MIKC-type MADS-domain TFs by a mechanism which is similar to the phenomenon called “latent specificity” (Joshi et al. 2007; Merabet and Mann 2016; Slattery et al. 2011). Latent specificity describes a protein–protein interaction between a transcription factor and a cofactor that uncovers a novel DNA-binding specificity (Merabet and Mann 2016). Here, the obligatory dimerization of MADS-domain proteins (Schwarz-Sommer et al. 1990) may provide this protein–protein interaction that modifies DNA-binding specificity.

It seems clear from this study and from our previous study (Käppel et al. 2018) that R3 is important for the DNA-binding preference for A/T-rich sequences and among these for (long) A-tract sequences. In this study, we have suggested that the arginine 3 residue of SEP3 recognizes the borders of the AT-rich CArG-box core (positions 3 and 8). As can be already seen from the comparison of SEP3 with MEF2A and SRF, the bases contacted by arginine 3 can vary between MADS-domain proteins (Fig. 5), although the MADS-domain and arginine 3 are highly conserved. This means that the high conservation of arginine 3 in MADS-domain proteins does not mean that the R3-DNA contacts are conserved. If one considers that MIKC-type MADS-domain TFs bind to DNA as homo- and heterodimers in different combinations, it is feasible that arginine 3 may be positioned differently within the minor groove and might make different base contacts depending on the protein dimer composition. The positioning of R3, which is determined by the MADS-domain protein dimer structure, seems to be crucial for the DNA-binding specificity of MADS-domain transcription factors.

### Comparison with previously published SELEX-seq data for SEP3

In a previous SELEX-seq study (Smaczniak et al. 2017b) SEP3 homodimers were found to bind to two different subtypes of CArG-boxes: the 10-base-pair motif 5′-CYATAAATRG-3′ and the 11-base-pair motif 5′-CYATAAATAGG-3′ (strand orientation was changed for easier comparison). The former motif is very similar to the binding motif, that we have found (5′-CCNWAAATGG-3′). We did not discover the latter motif during the de novo discovery, as the algorithm did always return the 10-base-pair motif even when searching for a larger pattern.

The preferred flanking sequences, that we have identified (5′-TTN- and -NAA-3′), are exactly the same as described in the SELEX-seq study performed by Smaczniak et al. In our study we were also interested in identifying dependencies between the CArG-box core and the flanking sequences, which we thought might exist in order to create certain DNA shapes over the complete sequence motif, which seems to be roughly 16 base pairs long. However, our dependency study revealed that such dependencies are barely detectable.

Whereas no clear differences between CArG-box sequences bound by different MADS-domain TFs in *A. thaliana* could be detected in ChIP-seq data (Aerts et al. 2018), the aforementioned SELEX-seq study (Smaczniak et al. 2017b) could discriminate CArG-boxes bound by e.g. SEP3 homodimers from sequences bound by AG (AGAMOUS) homodimers or from motifs bound by SEP3-AG heterodimers. They found for example that the A/T-rich CArG-box core was 3 to 5 base pairs long for the AG homodimer, 6 to 8 base pairs long for the SEP3 homodimer and 4 to 7 base pairs long for the SEP3-AG heterodimer. These differences might be accounted for by different interactions of the N-terminal arm of the MADS-domain with the target DNA. Since arginine 3 makes base contacts within the A/T-rich CArG-box core, it is likely involved in the readout of the A/T-rich sequence (or A-tract) length. Therefore, the different positioning of arginine 3 in these different protein dimers might be an important sensor to differentiate CArG-box subtypes with varying A-tract lengths and thus distinct binding motifs for different protein dimers. This hypothesis will be able to be tested when hopefully in the future 3D structures of plant MADS-domain proteins will be available.

## Conclusion

In this study, we extended our knowledge of the binding specificity of the MADS-domain transcription factor SEPALLATA3 with respect to the CArG-box flanking regions and concerning the role of arginine 3. We used SELEX-seq to get an overview of the DNA binding repertoire of SEP3 dimers.

Analysis of the 10mers with highest binding affinity yielded CArG-boxes and CArG-box-like sequences as was expected for a MADS-domain transcription factor. Closer analysis of the central positions showed stretches of three to six adenines or thymines (depending on strand alignment). We then confirmed that there is a binding preference for A-tract-containing CArG-boxes versus CArG-boxes without an A-tract.

In our previous work, we have demonstrated that the flanking sequences have a big influence on binding affinity of SEP3_MI_. In our in vitro experiments we had found differences in binding affinity up to the factor of 50 depending on the identity of the six nucleotides flanking the CArG-box on each side (Käppel et al. 2018). Here, we found that T- and A-rich sequences are preferred flanking sequences at the “CC” and the “GG” end, respectively. These “NAA extensions” have recently been found also in ChIP-seq data of MADS-domain proteins including SEPALLATA3 (Aerts et al. 2018) and in SELEX-seq data of different MADS-domain protein complexes including SEP3 homodimers (Smaczniak et al. 2017b).

Surprisingly, we found only weak dependencies between the CArG-box center and the flanking sequences. We had expected to find some dependencies because they had been reported in binding data for SEP3 coming from ChIP-seq experiments (Kaufmann et al. 2009).

We improved our understanding of the role of arginine residue R3 of the DNA-binding MADS-domain for binding specificity. Mutant studies revealed that this highly conserved amino acid residue is critical for the recognition of nucleotides at positions 3 and 8 of the CArG-box motif. Whereas, the wildtype SEP3_MI_ protein preferentially binds to CArG-boxes, which have an A/T-rich center with a length of six base pairs, the mutant proteins bind CArG-box sequences with a shorter A/T-rich sequence motif of only four base pairs. Our proposed DNA-binding mode of SEP3 is not identical to previously reported protein–DNA-complexes of the MADS-domain transcription factors SRF (Pellegrini et al. 1995) or MEF2A (Huang et al. 2000; Santelli and Richmond 2000) with their cognate DNA-binding sites. Although SEP3 binds to the SRF-type CArG-box, the proposed base contacts of SEP3 are more similar to the ones seen for MEF2A. Although arginine 3 is highly conserved, it seems to be important for DNA-binding specificity within the MADS-domain TF family since it is positioned differently within the minor groove depending on the MADS-domain protein dimer composition.

MADS-domain proteins achieve target gene specificity by a complex combination of different strategies: DNA‐binding specificity, protein dimerization, tetramer formation and the preference for certain distances between two DNA-binding sites, which are required for tetramer binding (Jetha et al. 2014; Kaufmann et al. 2005b; Melzer et al. 2006; Theißen et al. 2016). Not all of these facets could be addressed by the present study. For the future, in vivo experiments (e.g. ChIP-seq) using SEP3 mutant plants might be interesting to validate the importance of arginine 3 for DNA-binding specificity and to better understand the *in planta* relevance of our findings. If arginine 3 is crucial for DNA-binding, we would expect to see a disturbed flower development in SEP3 mutant plants.

## Supplementary Information

Below is the link to the electronic supplementary material.

(JPG 433 kb) **Supplementary Fig. S1** Probe design.** a** DNA probes were designed to include a central random region of 25 nucleotides (N_25_, marked in black), a probe barcode of 4 nt (red color) and flanking regions which are compatible with Illumina sequencing (TruSeq adapter sequences). The 5′-adapter (light green) contains the sequence of the sequencing primer, whereas the 3′-adapter (blue) contains the sequence of the index sequencing primer. The sequencing primers are also depicted by arrows. The nucleotides marked in dark green already belong to the 5′ Illumina Adapter (P5) which was added later. Full-length oligonucleotides were 99 nucleotides long and represented the forward strand. **b** Forward oligonucleotides were annealed with the oligonucleotide “Primer_SELEX_rev”. Second strand synthesis was done with Klenow Fragment (Thermo Fisher Scientific). DNA from the selection rounds was PCR amplified using the primers “Primer_SELEX_fwd” and “Primer_SELEX_rev” (Supplementary Table S2). **c** Sequencing libraries were finally generated by limited cycle PCR. In this step the Illumina Adapters P5 (dark green) and P7 (dark blue) and the 6-nucleotide-long cycle barcodes were added to the libraries. The primers, which were used, were “Primer_adapter_fwd” and “Primer_adapter_rev_0X”. “X” was dependent on the cycle and represented the cycle barcode (sequences are listed in Supplementary Table S2). **d** Sequencing libraries contained the 5′ Illumina Adapter P5 (dark green), the sequence for the annealing of the sequencing primer (light green), the random sequence N_25_ (black), the protein-specific probe barcode (red), the sequence for the annealing of the index sequencing primer (light blue), the cycle barcode (orange) and the 3′ Illumina Adapter P7 (dark blue). Sequencing libraries are listed in Supplementary Table S3

(JPG 1312 kb) **Supplementary Fig. S2** Enrichment of SEP3_MI_-bound DNA by 4 rounds of SELEX based on a gel shift assay. **a** The wildtype (WT) SEP3_MI_ protein was incubated with the DNA probe 1 over four selection rounds. This is replicate 1 of SEP3_MI_ WT. **b** The SEP3_MI_ R3A mutant protein was incubated with the DNA probe 2. This is replicate 1 of SEP3_MI_ R3A. **c** The SEP3_MI_ R3K mutant protein was incubated with the DNA probe 3. This is replicate 1 of SEP3_MI_ R3K. **d** The wildtype (WT) SEP3_MI_ protein was incubated with the DNA probe 4. This is replicate 2 of SEP3_MI_ WT. **e** The SEP3_MI_ R3A mutant protein was incubated with the DNA probe 5. This is replicate 2 of SEP3_MI_ R3A. **f** The SEP3_MI_ R3K mutant protein was incubated with the DNA probe 6. This is replicate 2 of SEP3_MI_ R3K. The specified SEP3_MI_ protein (WT, R3A or R3K) was co-incubated with the specified DNA probe (1–6) over four selection rounds. “R1”, “R2”, “R3” or “R4” refer to the SELEX round 1, 2, 3 or 4, respectively. After incubation samples were then loaded onto polyacrylamide gels. These were stained with ethidium bromide after the gel run. DNA ladders (Thermo Scientific GeneRuler 50 bp DNA Ladder and Thermo Scientific O’RangeRuler 20 bp DNA Ladder) were used for size orientation. Free DNA probes with a length of 99 base pairs (bp) were visible as a band of about 100 bp. The protein-bound DNA was shifted to an apparent size of 150–200 bp compared with the DNA ladders. Bands were excised according to the apparent size of the shifted DNA band of the positive control. There was an increase in signal intensity for the fraction of protein-bound DNA from cycle R1 to cycle R4 representing an increase of SEP3_MI_-bound DNA with every cycle. Samples marked with an asterisk (most of the libraries of the amplification rounds 3 and 4) were found to be highly contaminated with the positive control. Highly contaminated samples were excluded from further analysis

(JPG 688 kb) **Supplementary Fig. S3** Limited cycle PCR was used to add the final adapters to the libraries. These PCR products were then gel-purified. The gel pictures only show the purification of libraries from round 0 (R0), i.e. the initial libraries. **a** PCR products were run on a 0.5 × TBE 5% polyacrylamide gel. After ethidium bromide staining the gel was placed on a transilluminator UV table (Appligene). DNA ladders (Thermo Scientific GeneRuler 50 bp DNA Ladder and Thermo Scientific O’RangeRuler 20 bp DNA Ladder) were used for size orientation of PCR products. **b** The band which ran at an apparent height of 150 bp (PCR product length with complete adapter sequences) was excised. The elution of DNA from the gel was done as previously described (Riley et al. 2014)

(JPG 688 kb) **Supplementary Fig. S4** Markov model optimization**.** To model the biases of the initial pools R0, the optimal order for the Markov model of the initial pools R0 were determined. We followed the protocol described by Riley et al. (2014). The optimization was done by quantifying how a Markov model trained on one replicate of R0 predicts 8-mer counts in another replicate in terms of a coefficient of determination (R2). A fourth- or a fifth-order model has the best cross-validation performance depending on the experiment

(JPG 688 kb) **Supplementary Fig. S5** Oligonucleotide length (K-mer) optimization. To determine the optimal motif length which should be used to calculate relative affinities, the information gain (Kullback–Leibler divergence) associated with two rounds of selection (from R0 to R2) was computed according to Riley et al. (2014). The optimal oligonucleotide length was determined as 10 base pairs with the exception of SEP3_MI_ R3A (Replicate 2)

(JPG 688 kb) **Supplementary Fig. S6** Determination of relative affinities. The normalized relative affinity values calculated from the enrichment from round R0 to round R1 and the normalized relative affinity values based on the enrichment between round R0 and round R2 can be compared in these scatter plots. The deviation from the straight line might be due to a combination of different effects, e.g. PCR bias

(JPG 688 kb) **Supplementary Fig. S7** Determination of relative affinities with LOESS regression. Information from multiple rounds of SELEX selection was integrated by using LOESS regression as has been previously described (Riley et al 2014). R1-based affinity estimates and LOESS-based estimates resulting from integration of R1 and R2 data can be compared in these scatter plots. There is a linear relationship between these values

(JPG 688 kb) **Supplementary Fig. S8** Comparison of the location of complete and incomplete CArG- boxes within the variable 25 bp segment of the DNA probe. Incomplete CArG-boxes are here defined as being one of the top 100 k-mers according to affinity score and matching (A/T)_2_(N)_6_(A/T)_2_. They occur predominantly at position 1 [if matching (A/T)_6_GG(A/T)_2_] and position 2 [if matching (A/T)_5_GG(A/T)_3_]. In both cases the terminal CT dinucleotide of the Illumina sequencing adapter appears to serve as 5′-end of a nearly perfect SRF-type CArG-box

(JPG 688 kb) **Supplementary Fig. S9** Visualizations of models compared in motif complexity analysis. **a** Proximal dependence model of order 1. The probability of observing a nucleotide is allowed to depend on the observed nucleotide on the previous position in the sequence. Hence, there are up to four nucleotide stacks for each position, but possibly fewer when the conditional nucleotide distributions are either not sufficiently different from each other or some context symbols appear very infrequently (or not at all), see e.g. the motif core. This grouping of context symbols is learned from data and represented by a context tree. The difference among the nucleotide stacks among a position are visible, but comparatively small and limited to small variations without a change in the consensus nucleotide. **b** Proximal dependence model of order 2. The visualization is similar, except that the context is now up to two nucleotides long, and hence the context trees have two layers. The first layer represents the directly preceding nucleotide. We observe a minor refinement of the first-order model without dramatic changes. **c** and **d** Distal dependence of order 1 and 2. Here, the requirement of having dependencies constrained to directly preceding nucleotides is dropped. The general dependence structure, that is, the decision which other positions a particular nucleotide distribution is conditioned upon, is learned from data and visualized on top, where an arc from position i to j indicates that j is conditioned on i. Here, most dependencies occur among neighboring nucleotides and there are no strong dependencies between the motif center and the flanking regions

(JPG 688 kb) **Supplementary Fig. S10** Difference logos created with *DiffLogo* (Nettling et al. 2015). Note that the range of the values is nearly one order of magnitude smaller than in Fig. 4, so all differences are very small in absolute terms. **a**–**c** Replicates 1 and 2 of the three protein variants are being compared. Differences are especially low for the two replicates of the wildtype protein (**a**). **d** and **e** show that there are no big differences in the DNA binding motifs of SEP3_MI_ R3A and SEP3_MI_ R3K

(DOCX 688 kb)

## Data Availability

The sequencing data was stored in the Sequence Read Archive (SRA) of the National Center for Biotechnology Information (NCBI). The SRA accession Number PRJNA609240.
