# Supplementary figures and images for: DNA-binding properties of the MADS-domain transcription factor SEPALLATA3 and mutant variants characterized by SELEX-seq
Source: Plant Mol Biol. 2021 Jan 24;105(4):543–57. doi: 10.1007/s11103-020-01108-6 (PMC7892521; doi:10.1007/s11103-020-01108-6)

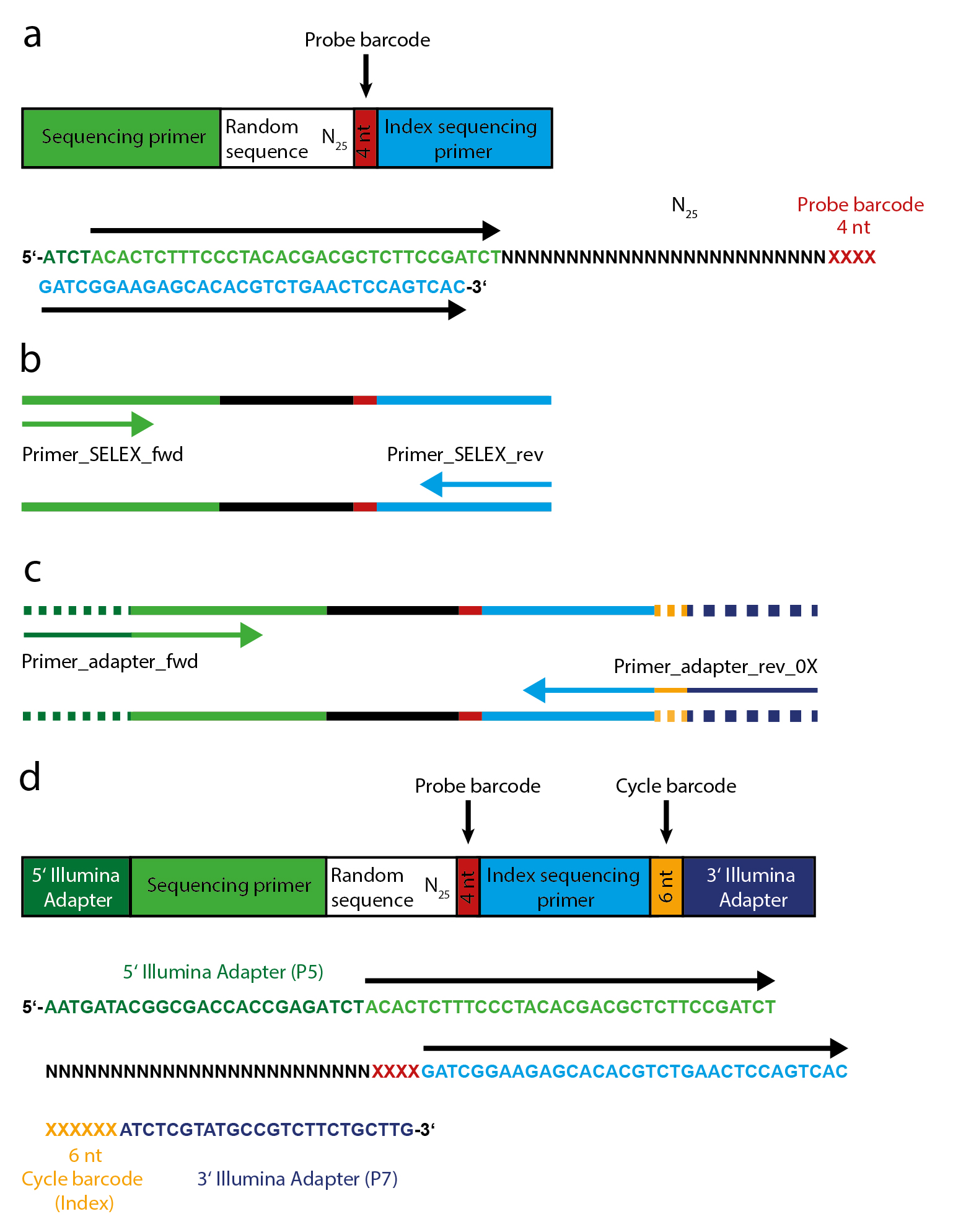

Supplement: Supplementary file 1 — (JPG 433 kb) Supplementary Fig. S1 Probe design. a DNA probes were designed to include a central random region of 25 nucleotides (N25, marked in black), a probe barcode of 4 nt (red color) and flanking regions which are compatible with Illumina sequencing (TruSeq adapter sequences). The 5′-adapter (light green) contains the sequence of the sequencing primer, whereas the 3′-adapter (blue) contains the sequence of the index sequencing primer. The sequencing primers are also depicted by arrows. The nucleotides marked in dark green already belong to the 5′ Illumina Adapter (P5) which was added later. Full-length oligonucleotides were 99 nucleotides long and represented the forward strand. b Forward oligonucleotides were annealed with the oligonucleotide “Primer_SELEX_rev”. Second strand synthesis was done with Klenow Fragment (Thermo Fisher Scientific). DNA from the selection rounds was PCR amplified using the primers “Primer_SELEX_fwd” and “Primer_SELEX_rev” (Supplementary Table S2). c Sequencing libraries were finally generated by limited cycle PCR. In this step the Illumina Adapters P5 (dark green) and P7 (dark blue) and the 6-nucleotide-long cycle barcodes were added to the libraries. The primers, which were used, were “Primer_adapter_fwd” and “Primer_adapter_rev_0X”. “X” was dependent on the cycle and represented the cycle barcode (sequences are listed in Supplementary Table S2). d Sequencing libraries contained the 5′ Illumina Adapter P5 (dark green), the sequence for the annealing of the sequencing primer (light green), the random sequence N25 (black), the protein-specific probe barcode (red), the sequence for the annealing of the index sequencing primer (light blue), the cycle barcode (orange) and the 3′ Illumina Adapter P7 (dark blue). Sequencing libraries are listed in Supplementary Table S3 [file 11103_2020_1108_MOESM1_ESM.jpg]

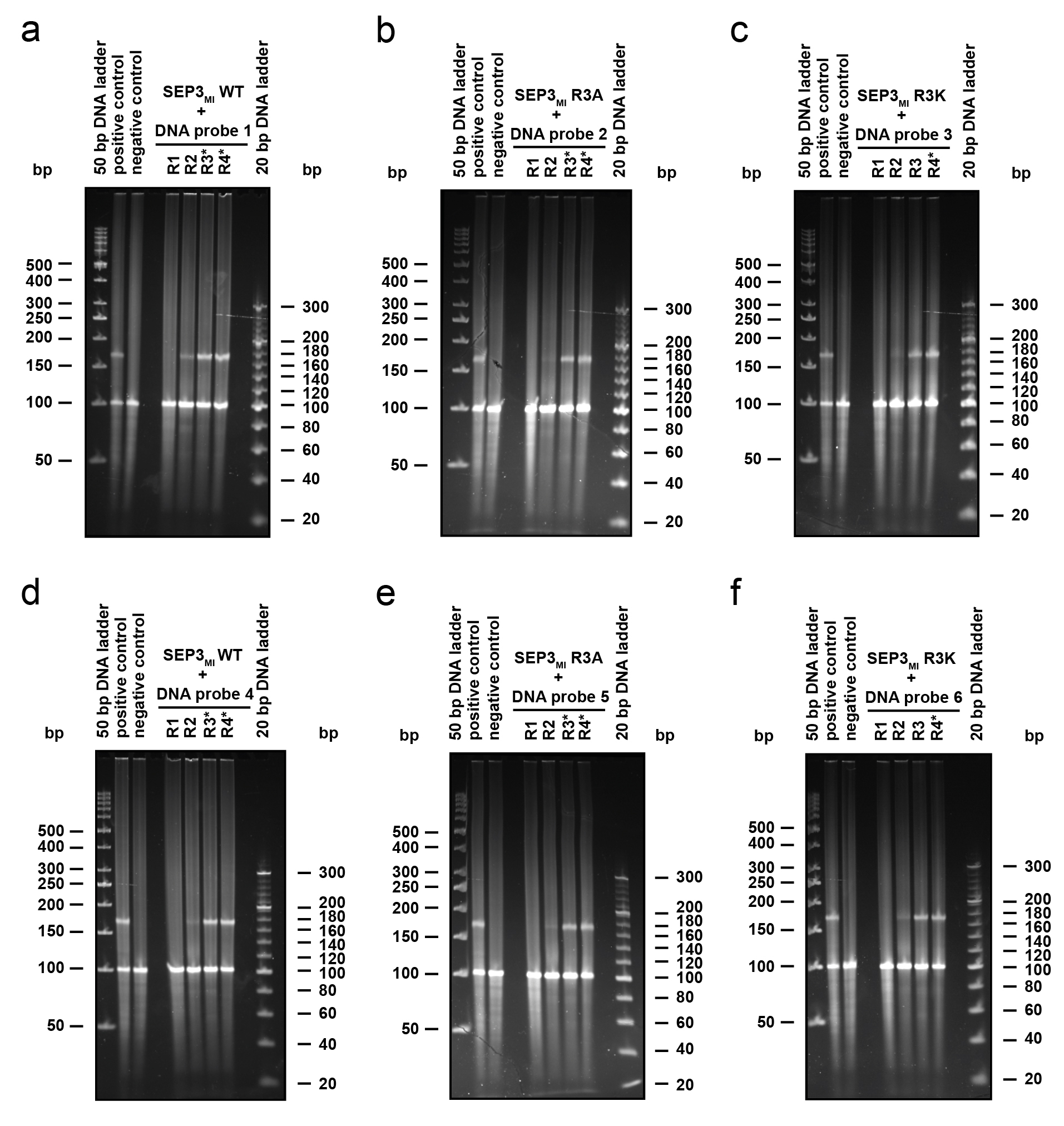

Supplement: Supplementary file 2 — (JPG 1312 kb) Supplementary Fig. S2 Enrichment of SEP3MI-bound DNA by 4 rounds of SELEX based on a gel shift assay. a The wildtype (WT) SEP3MI protein was incubated with the DNA probe 1 over four selection rounds. This is replicate 1 of SEP3MI WT. b The SEP3MI R3A mutant protein was incubated with the DNA probe 2. This is replicate 1 of SEP3MI R3A. c The SEP3MI R3K mutant protein was incubated with the DNA probe 3. This is replicate 1 of SEP3MI R3K. d The wildtype (WT) SEP3MI protein was incubated with the DNA probe 4. This is replicate 2 of SEP3MI WT. e The SEP3MI R3A mutant protein was incubated with the DNA probe 5. This is replicate 2 of SEP3MI R3A. f The SEP3MI R3K mutant protein was incubated with the DNA probe 6. This is replicate 2 of SEP3MI R3K. The specified SEP3MI protein (WT, R3A or R3K) was co-incubated with the specified DNA probe (1–6) over four selection rounds. “R1”, “R2”, “R3” or “R4” refer to the SELEX round 1, 2, 3 or 4, respectively. After incubation samples were then loaded onto polyacrylamide gels. These were stained with ethidium bromide after the gel run. DNA ladders (Thermo Scientific GeneRuler 50 bp DNA Ladder and Thermo Scientific O’RangeRuler 20 bp DNA Ladder) were used for size orientation. Free DNA probes with a length of 99 base pairs (bp) were visible as a band of about 100 bp. The protein-bound DNA was shifted to an apparent size of 150–200 bp compared with the DNA ladders. Bands were excised according to the apparent size of the shifted DNA band of the positive control. There was an increase in signal intensity for the fraction of protein-bound DNA from cycle R1 to cycle R4 representing an increase of SEP3MI-bound DNA with every cycle. Samples marked with an asterisk (most of the libraries of the amplification rounds 3 and 4) were found to be highly contaminated with the positive control. Highly contaminated samples were excluded from further analysis [file 11103_2020_1108_MOESM2_ESM.jpg]

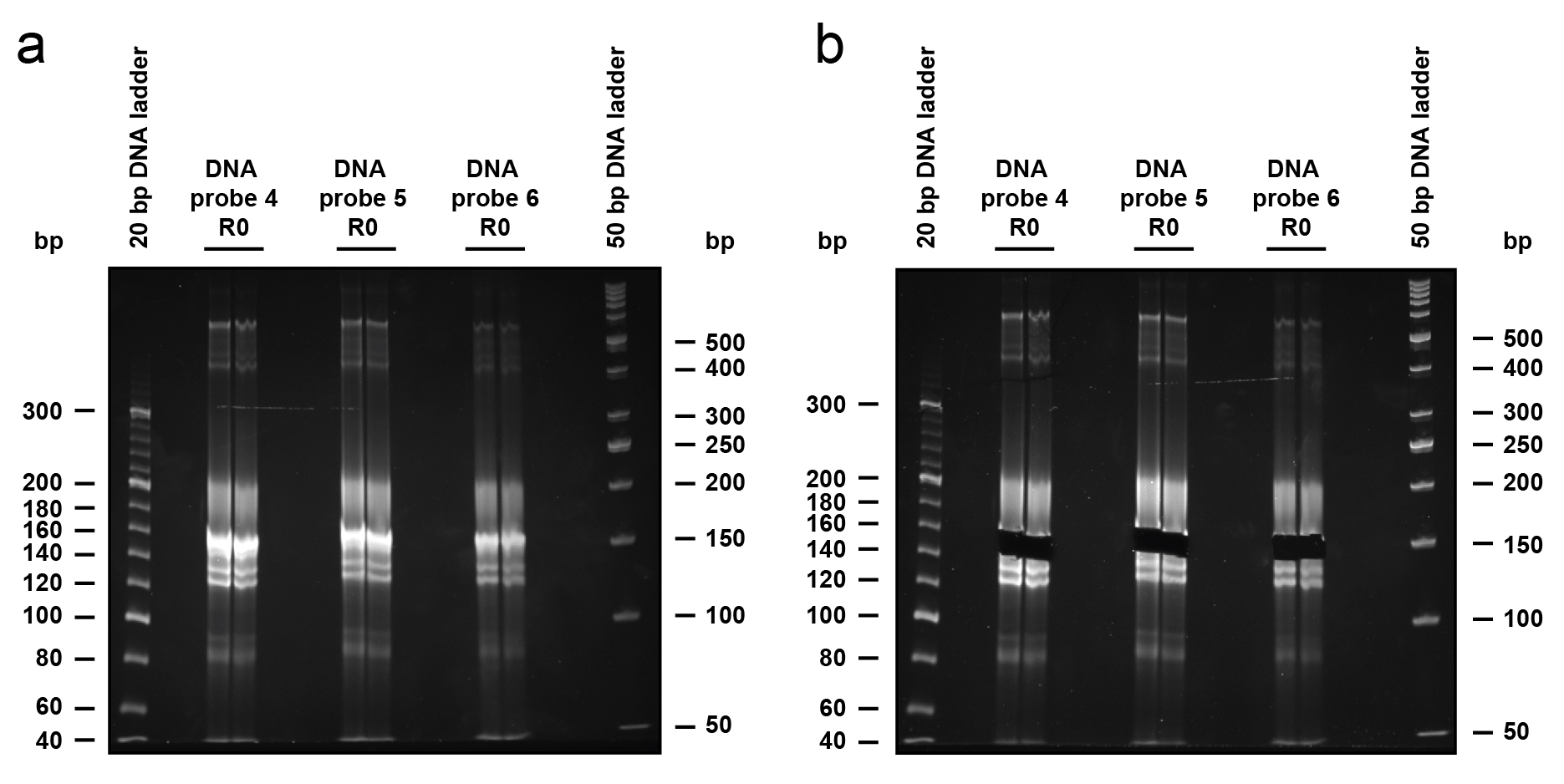

Supplement: Supplementary file 3 — (JPG 688 kb) Supplementary Fig. S3 Limited cycle PCR was used to add the final adapters to the libraries. These PCR products were then gel-purified. The gel pictures only show the purification of libraries from round 0 (R0), i.e. the initial libraries. a PCR products were run on a 0.5 × TBE 5% polyacrylamide gel. After ethidium bromide staining the gel was placed on a transilluminator UV table (Appligene). DNA ladders (Thermo Scientific GeneRuler 50 bp DNA Ladder and Thermo Scientific O’RangeRuler 20 bp DNA Ladder) were used for size orientation of PCR products. b The band which ran at an apparent height of 150 bp (PCR product length with complete adapter sequences) was excised. The elution of DNA from the gel was done as previously described (Riley et al. 2014) [file 11103_2020_1108_MOESM3_ESM.jpg]

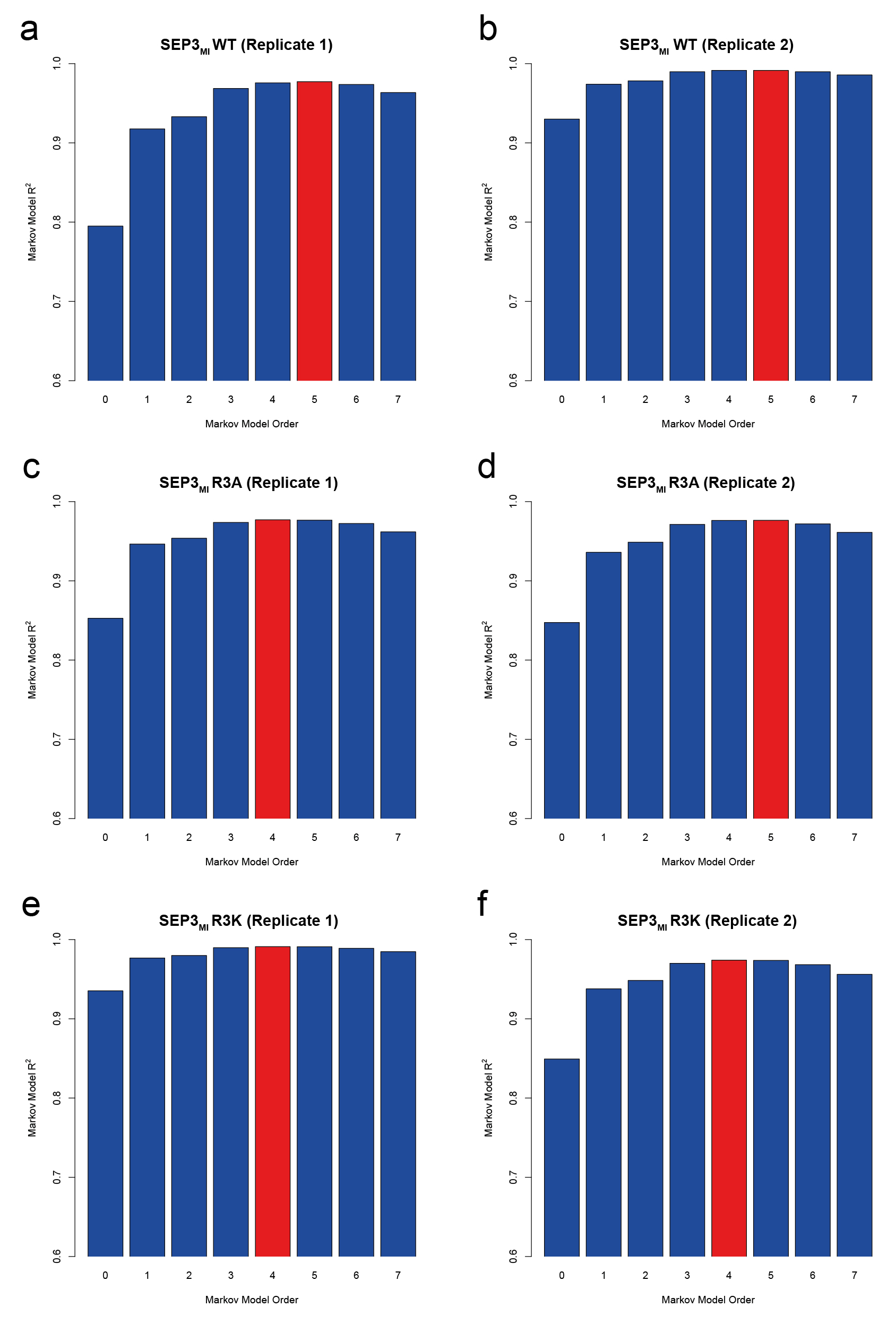

Supplement: Supplementary file 4 — (JPG 688 kb) Supplementary Fig. S4 Markov model optimization. To model the biases of the initial pools R0, the optimal order for the Markov model of the initial pools R0 were determined. We followed the protocol described by Riley et al. (2014). The optimization was done by quantifying how a Markov model trained on one replicate of R0 predicts 8-mer counts in another replicate in terms of a coefficient of determination (R2). A fourth- or a fifth-order model has the best cross-validation performance depending on the experiment [file 11103_2020_1108_MOESM4_ESM.jpg]

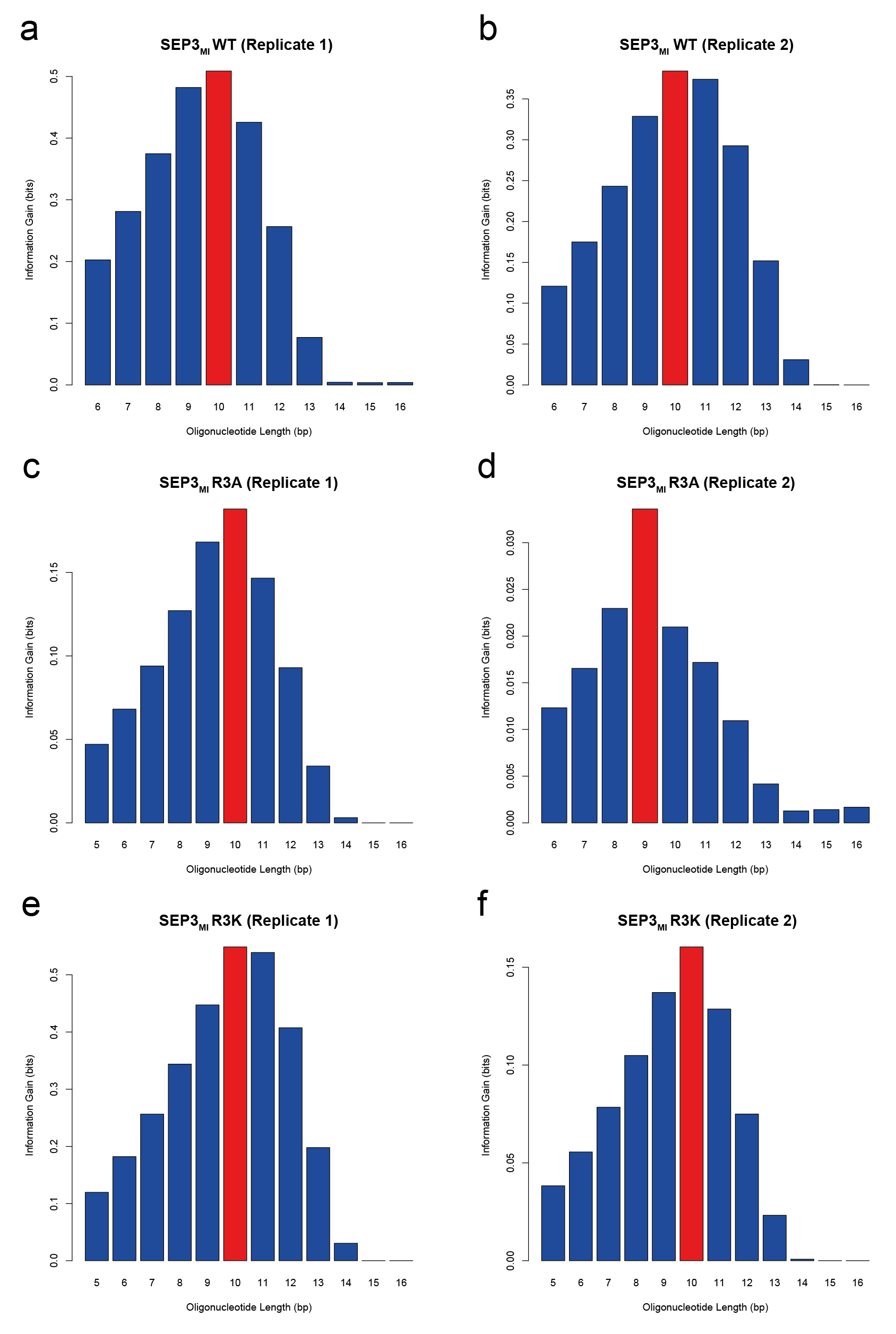

Supplement: Supplementary file 5 — (JPG 688 kb) Supplementary Fig. S5 Oligonucleotide length (K-mer) optimization. To determine the optimal motif length which should be used to calculate relative affinities, the information gain (Kullback–Leibler divergence) associated with two rounds of selection (from R0 to R2) was computed according to Riley et al. (2014). The optimal oligonucleotide length was determined as 10 base pairs with the exception of SEP3MI R3A (Replicate 2) [file 11103_2020_1108_MOESM5_ESM.jpg]

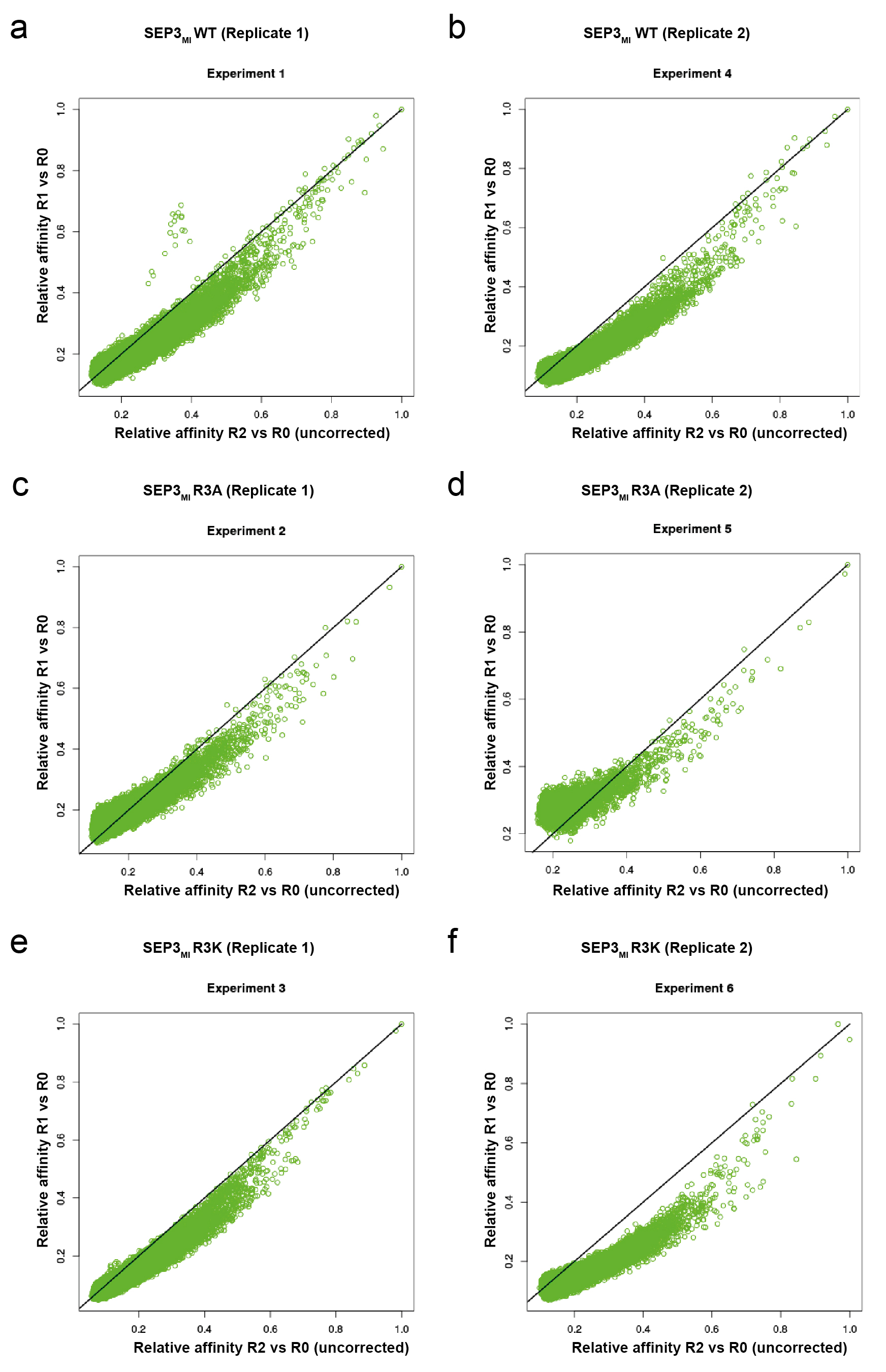

Supplement: Supplementary file 6 — (JPG 688 kb) Supplementary Fig. S6 Determination of relative affinities. The normalized relative affinity values calculated from the enrichment from round R0 to round R1 and the normalized relative affinity values based on the enrichment between round R0 and round R2 can be compared in these scatter plots. The deviation from the straight line might be due to a combination of different effects, e.g. PCR bias [file 11103_2020_1108_MOESM6_ESM.jpg]

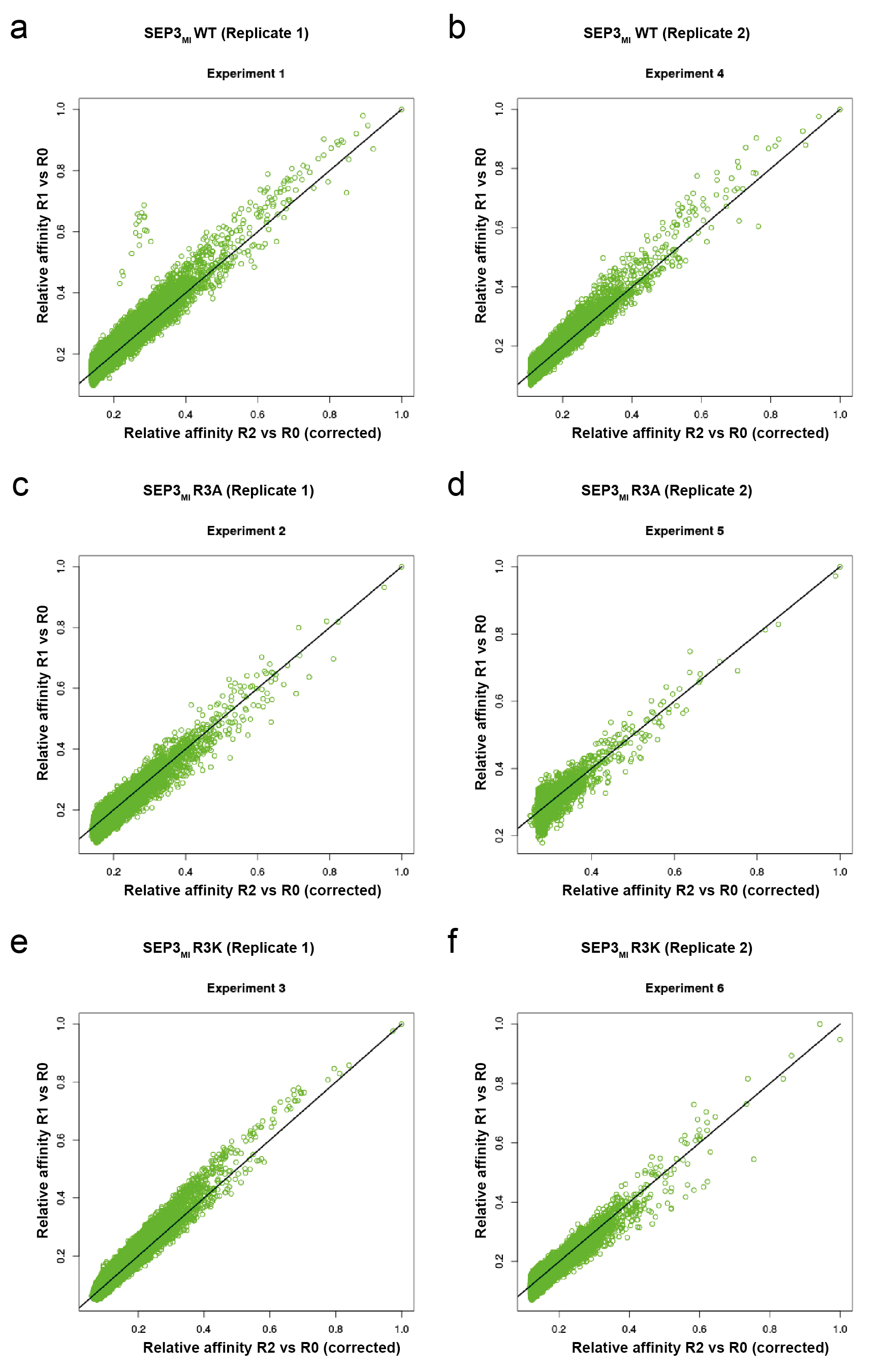

Supplement: Supplementary file 7 — (JPG 688 kb) Supplementary Fig. S7 Determination of relative affinities with LOESS regression. Information from multiple rounds of SELEX selection was integrated by using LOESS regression as has been previously described (Riley et al 2014). R1-based affinity estimates and LOESS-based estimates resulting from integration of R1 and R2 data can be compared in these scatter plots. There is a linear relationship between these values [file 11103_2020_1108_MOESM7_ESM.jpg]

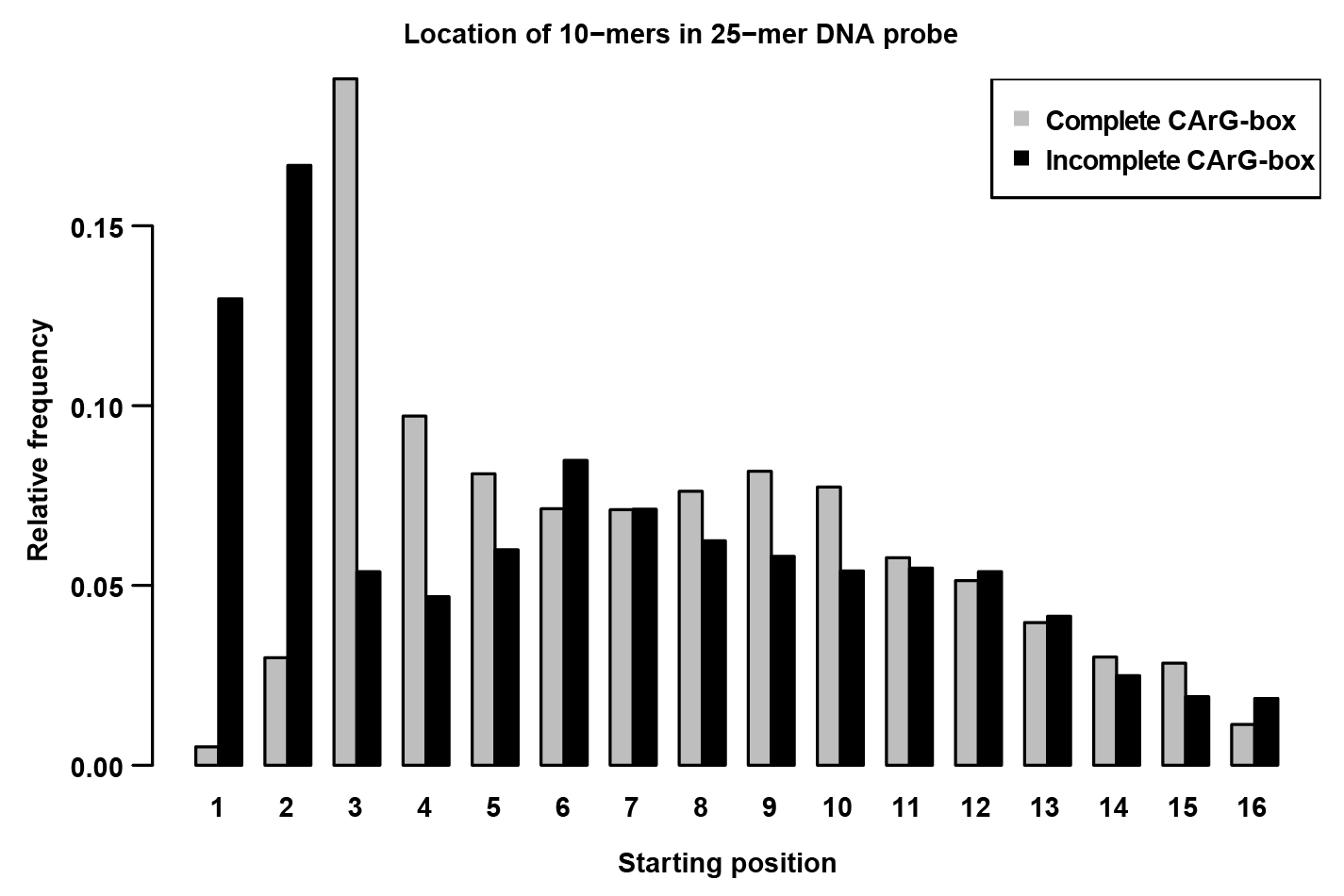

Supplement: Supplementary file 8 — (JPG 688 kb) Supplementary Fig. S8 Comparison of the location of complete and incomplete CArG- boxes within the variable 25 bp segment of the DNA probe. Incomplete CArG-boxes are here defined as being one of the top 100 k-mers according to affinity score and matching (A/T)2(N)6(A/T)2. They occur predominantly at position 1 [if matching (A/T)6GG(A/T)2] and position 2 [if matching (A/T)5GG(A/T)3]. In both cases the terminal CT dinucleotide of the Illumina sequencing adapter appears to serve as 5′-end of a nearly perfect SRF-type CArG-box [file 11103_2020_1108_MOESM8_ESM.jpg]

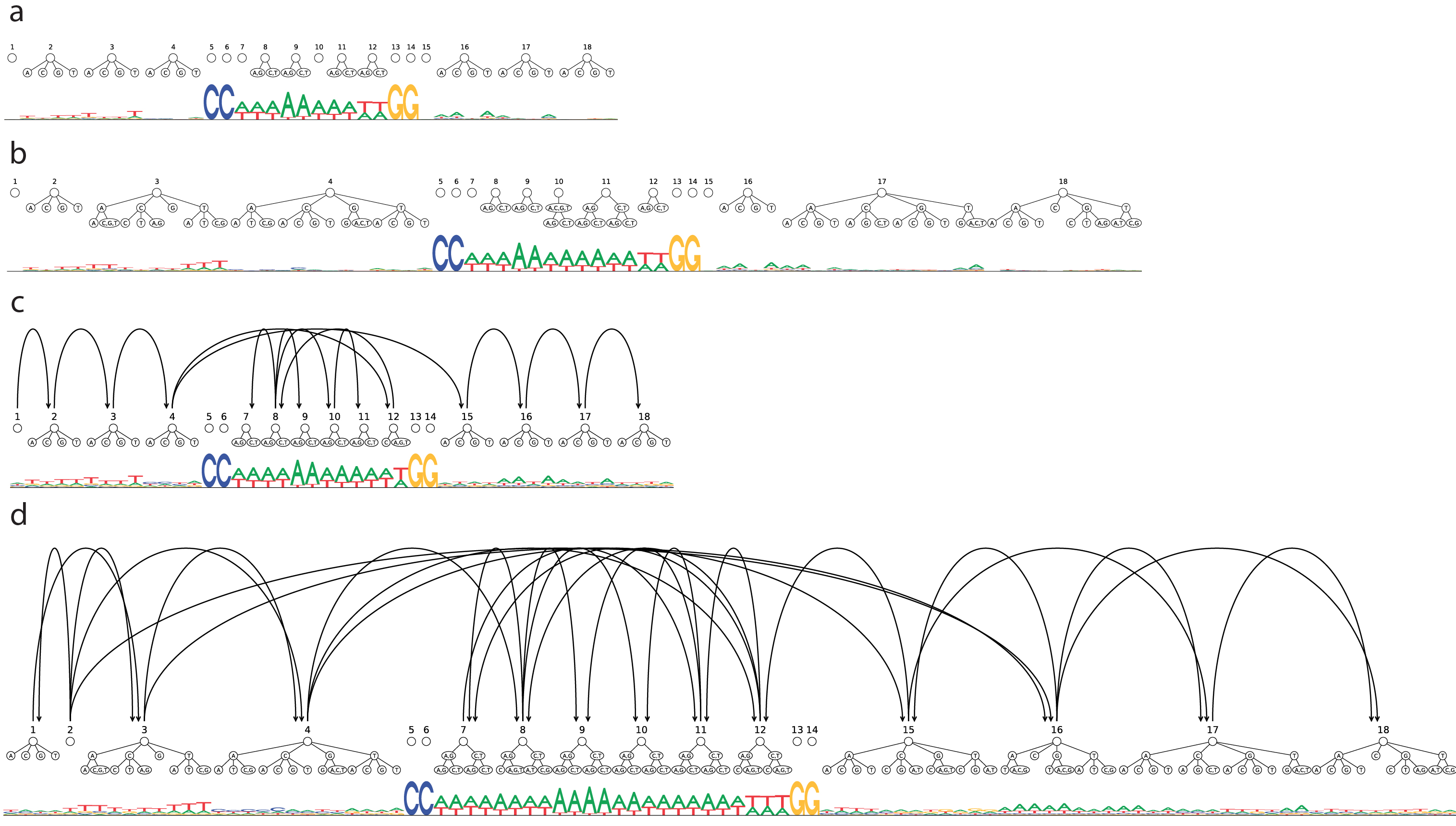

Supplement: Supplementary file 9 — (JPG 688 kb) Supplementary Fig. S9 Visualizations of models compared in motif complexity analysis. a Proximal dependence model of order 1. The probability of observing a nucleotide is allowed to depend on the observed nucleotide on the previous position in the sequence. Hence, there are up to four nucleotide stacks for each position, but possibly fewer when the conditional nucleotide distributions are either not sufficiently different from each other or some context symbols appear very infrequently (or not at all), see e.g. the motif core. This grouping of context symbols is learned from data and represented by a context tree. The difference among the nucleotide stacks among a position are visible, but comparatively small and limited to small variations without a change in the consensus nucleotide. b Proximal dependence model of order 2. The visualization is similar, except that the context is now up to two nucleotides long, and hence the context trees have two layers. The first layer represents the directly preceding nucleotide. We observe a minor refinement of the first-order model without dramatic changes. c and d Distal dependence of order 1 and 2. Here, the requirement of having dependencies constrained to directly preceding nucleotides is dropped. The general dependence structure, that is, the decision which other positions a particular nucleotide distribution is conditioned upon, is learned from data and visualized on top, where an arc from position i to j indicates that j is conditioned on i. Here, most dependencies occur among neighboring nucleotides and there are no strong dependencies between the motif center and the flanking regions [file 11103_2020_1108_MOESM9_ESM.jpg]

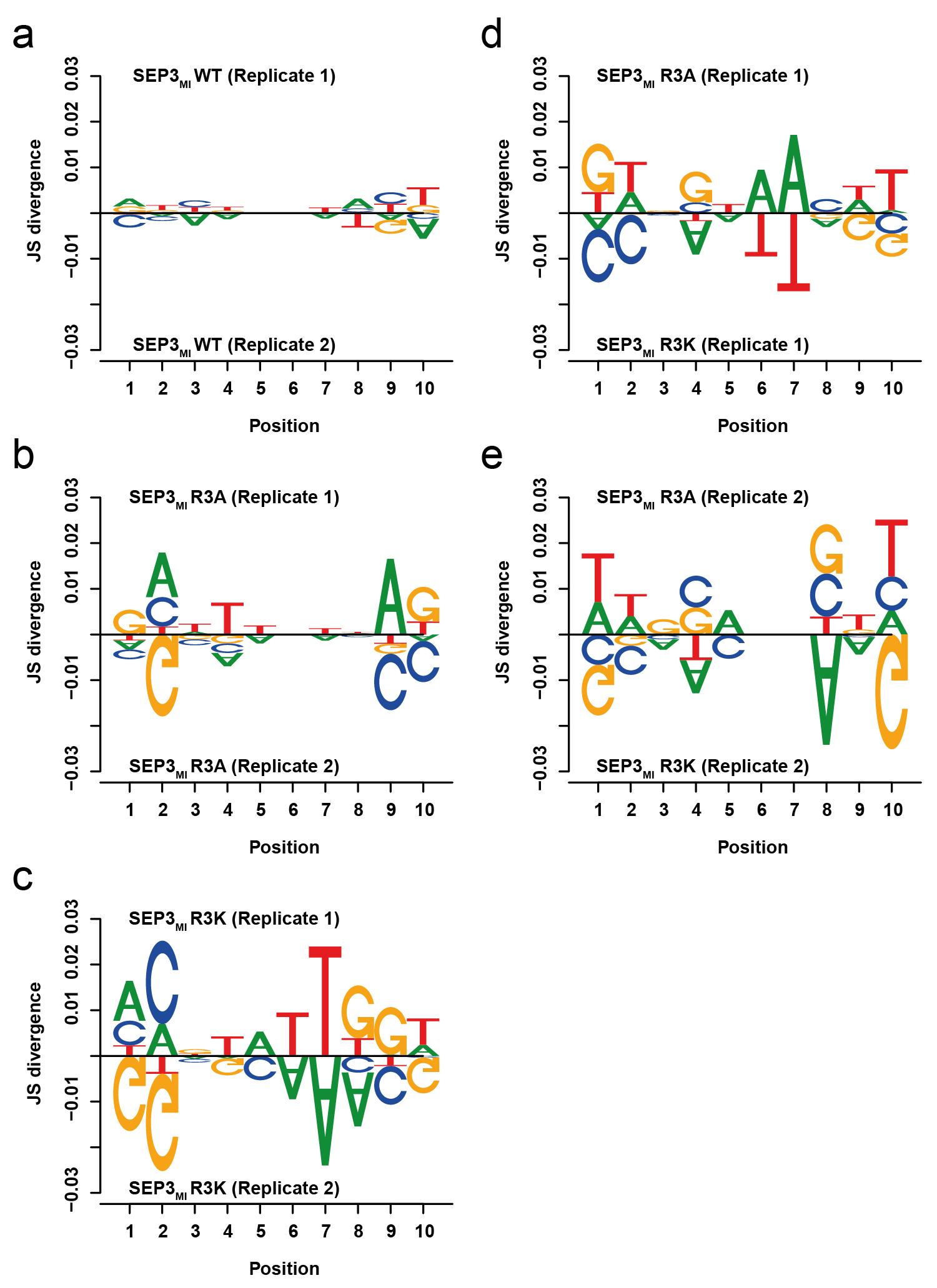

Supplement: Supplementary file 10 — (JPG 688 kb) Supplementary Fig. S10 Difference logos created with DiffLogo (Nettling et al. 2015). Note that the range of the values is nearly one order of magnitude smaller than in Fig. 4, so all differences are very small in absolute terms. a–c Replicates 1 and 2 of the three protein variants are being compared. Differences are especially low for the two replicates of the wildtype protein (a). d and e show that there are no big differences in the DNA binding motifs of SEP3MI R3A and SEP3MI R3K [file 11103_2020_1108_MOESM10_ESM.jpg]
