# Supplementary material for: DNA-binding properties of the MADS-domain transcription factor SEPALLATA3 and mutant variants characterized by SELEX-seq
Source: Plant Mol Biol. 2021 Jan 24;105(4):543–57. doi: 10.1007/s11103-020-01108-6 (PMC7892521; doi:10.1007/s11103-020-01108-6)
Supplement: Supplementary file 11 — (DOCX 688 kb) [file 11103_2020_1108_MOESM11_ESM.docx]

**Supplementary Table S1**. Sequences of DNA probes for the gel shifts of the SELEX protocol. Only the sequences of the forward strand are given. The reverse strand was synthesized by primer extension using the reverse primer “Primer_SELEX_rev” (Supplementary Table S2) and DNA Polymerase I, Large (Klenow) Fragment. The probe number barcode is underlined. For the positive control the sequence of the CArG box 1 of the *AG* intron 2 (marked in bold) was used (first described by Hong et al., 2003; shown to be bound by SEP3 *in* *vitro* by Kaufmann et al., 2005). For the negative control the same sequence as for the positive control was used except for the fact that the CArG-box was mutated (marked in red).

| **Probe name** | **Sequence 5’-3’** |
| --- | --- |
| **EMSA_probe_1** | ATCTACACTCTTTCCCTACACGACGCTCTTCCGATCTNNNNNNNNNNNNNNNNNNNNNNNNNCTGAGATCGGAAGAGCACACGTCTGAACTCCAGTCAC |
| **EMSA_probe_2** | ATCTACACTCTTTCCCTACACGACGCTCTTCCGATCTNNNNNNNNNNNNNNNNNNNNNNNNNGACTGATCGGAAGAGCACACGTCTGAACTCCAGTCAC |
| **EMSA_probe_3** | ATCTACACTCTTTCCCTACACGACGCTCTTCCGATCTNNNNNNNNNNNNNNNNNNNNNNNNNACTGGATCGGAAGAGCACACGTCTGAACTCCAGTCAC |
| **EMSA_probe_4** | ATCTACACTCTTTCCCTACACGACGCTCTTCCGATCTNNNNNNNNNNNNNNNNNNNNNNNNNTGACGATCGGAAGAGCACACGTCTGAACTCCAGTCAC |
| **EMSA_probe_5** | ATCTACACTCTTTCCCTACACGACGCTCTTCCGATCTNNNNNNNNNNNNNNNNNNNNNNNNNCGATGATCGGAAGAGCACACGTCTGAACTCCAGTCAC |
| **EMSA_probe_6** | ATCTACACTCTTTCCCTACACGACGCTCTTCCGATCTNNNNNNNNNNNNNNNNNNNNNNNNNTACGGATCGGAAGAGCACACGTCTGAACTCCAGTCAC |
| **EMSA_probe_positive** | ATCTACACTCTTTCCCTACACGACGCTCTTCCGATCT**TTATATTCCAAATAAGGAAAGTATG**CATGGATCGGAAGAGCACACGTCTGAACTCCAGTCAC |
| **EMSA_probe_negative** | ATCTACACTCTTTCCCTACACGACGCTCTTCCGATCT**TTATATTGGAAATAACCAAAGTATG**AGCTGATCGGAAGAGCACACGTCTGAACTCCAGTCAC |

**Supplementary Table S2.** List of PCR primers.

| Oligonucleotide name | Sequence 5’-3’ |
| --- | --- |
| PCR amplification rounds | |
| Primer_SELEX_fwd | ATCTACACTCTTTCCCTACACGACGC |
| Primer_SELEX_rev | GTGACTGGAGTTCAGACGTGTGC |
| Adapter addition by limited cycle PCR (barcodes for SELEX cycle are underlined) | |
| Primer_adapter_fwd | AATGATACGGCGACCACCGAGATCTACACTCTTTCCCTACACGACGC |
| Primer_adapter_rev_00 | CAAGCAGAAGACGGCATACGAGATCAGTAGGTGACTGGAGTTCAGACGTGTGC |
| Primer_adapter_rev_01 | CAAGCAGAAGACGGCATACGAGATGGTGCAGTGACTGGAGTTCAGACGTGTGC |
| Primer_adapter_rev_02 | CAAGCAGAAGACGGCATACGAGATATACGCGTGACTGGAGTTCAGACGTGTGC |
| Primer_adapter_rev_03 | CAAGCAGAAGACGGCATACGAGATTCCATGGTGACTGGAGTTCAGACGTGTGC |
| Primer_adapter_rev_04 | CAAGCAGAAGACGGCATACGAGATCTACGTGTGACTGGAGTTCAGACGTGTGC |

**Supplementary Table S3.** List of SELEX sequencing libraries. After the gel shift assay the final adapters for Illumina sequencing were added via PCR. In the second column the probe number and the protein, which had been added for the respective protein-DNA binding reactions, are given. The complete sequences include all adapters and all barcodes. For simplicity only the forward sequence is given. Barcodes representing the probe number (4 nucleotides) are underlined and barcodes representing the SELEX cycle (6 nucleotides) are both underlined and in italics.

| **SELEX library name** | **Probe number/**  **Protein added** | **SELEX cycle** | **Sequence 5‘-3‘** |
| --- | --- | --- | --- |
| **1_R0 adapter** | 1  - | R0 | AATGATACGGCGACCACCGAGATCTACACTCTTTCCCTACACGACGCTCTTCCGATCTNNNNNNNNNNNNNNNNNNNNNNNNNCTGAGATCGGAAGAGCACACGTCTGAACTCCAGTCAC*CTACTG*ATCTCGTATGCCGTCTTCTGCTTG |
| **2_R0 adapter** | 2  - | R0 | AATGATACGGCGACCACCGAGATCTACACTCTTTCCCTACACGACGCTCTTCCGATCTNNNNNNNNNNNNNNNNNNNNNNNNNGACTGATCGGAAGAGCACACGTCTGAACTCCAGTCAC*CTACTG*ATCTCGTATGCCGTCTTCTGCTTG |
| **3_R0 adapter** | 3  - | R0 | AATGATACGGCGACCACCGAGATCTACACTCTTTCCCTACACGACGCTCTTCCGATCTNNNNNNNNNNNNNNNNNNNNNNNNNACTGGATCGGAAGAGCACACGTCTGAACTCCAGTCAC*CTACTG*ATCTCGTATGCCGTCTTCTGCTTG |
| **4_R0 adapter** | 4  - | R0 | AATGATACGGCGACCACCGAGATCTACACTCTTTCCCTACACGACGCTCTTCCGATCTNNNNNNNNNNNNNNNNNNNNNNNNNTGACGATCGGAAGAGCACACGTCTGAACTCCAGTCAC*CTACTG*ATCTCGTATGCCGTCTTCTGCTTG |
| **5_R0 adapter** | 5  - | R0 | AATGATACGGCGACCACCGAGATCTACACTCTTTCCCTACACGACGCTCTTCCGATCTNNNNNNNNNNNNNNNNNNNNNNNNNCGATGATCGGAAGAGCACACGTCTGAACTCCAGTCAC*CTACTG*ATCTCGTATGCCGTCTTCTGCTTG |
| **6_R0 adapter** | 6  - | R0 | AATGATACGGCGACCACCGAGATCTACACTCTTTCCCTACACGACGCTCTTCCGATCTNNNNNNNNNNNNNNNNNNNNNNNNNTACGGATCGGAAGAGCACACGTCTGAACTCCAGTCAC*CTACTG*ATCTCGTATGCCGTCTTCTGCTTG |
| **1_R1_WT adapter** | 1  SEP3_MI_ WT | R1 | AATGATACGGCGACCACCGAGATCTACACTCTTTCCCTACACGACGCTCTTCCGATCTNNNNNNNNNNNNNNNNNNNNNNNNNCTGAGATCGGAAGAGCACACGTCTGAACTCCAGTCAC*TGCACC*ATCTCGTATGCCGTCTTCTGCTTG |
| **2_R1_**  **R3A adapter** | 2  SEP3_MI_ R3A | R1 | AATGATACGGCGACCACCGAGATCTACACTCTTTCCCTACACGACGCTCTTCCGATCTNNNNNNNNNNNNNNNNNNNNNNNNNGACTGATCGGAAGAGCACACGTCTGAACTCCAGTCAC*TGCACC*ATCTCGTATGCCGTCTTCTGCTTG |
| **3_R1_**  **R3K adapter** | 3  SEP3_MI_ R3K | R1 | AATGATACGGCGACCACCGAGATCTACACTCTTTCCCTACACGACGCTCTTCCGATCTNNNNNNNNNNNNNNNNNNNNNNNNNACTGGATCGGAAGAGCACACGTCTGAACTCCAGTCAC*TGCACC*ATCTCGTATGCCGTCTTCTGCTTG |
| **4_R1_WT adapter** | 4  SEP3_MI_ WT | R1 | AATGATACGGCGACCACCGAGATCTACACTCTTTCCCTACACGACGCTCTTCCGATCTNNNNNNNNNNNNNNNNNNNNNNNNNTGACGATCGGAAGAGCACACGTCTGAACTCCAGTCAC*TGCACC*ATCTCGTATGCCGTCTTCTGCTTG |
| **Sample name** | **Probe number/**  **Protein added** | **SELEX cycle** | **Sequence 5‘-3‘** |
| **5_R1_ R3A adapter** | 5  SEP3_MI_ R3A | R1 | AATGATACGGCGACCACCGAGATCTACACTCTTTCCCTACACGACGCTCTTCCGATCTNNNNNNNNNNNNNNNNNNNNNNNNNCGATGATCGGAAGAGCACACGTCTGAACTCCAGTCAC*TGCACC*ATCTCGTATGCCGTCTTCTGCTTG |
| **6_R1_**  **R3K adapter** | 6  SEP3_MI_ R3K | R1 | AATGATACGGCGACCACCGAGATCTACACTCTTTCCCTACACGACGCTCTTCCGATCTNNNNNNNNNNNNNNNNNNNNNNNNNTACGGATCGGAAGAGCACACGTCTGAACTCCAGTCAC*TGCACC*ATCTCGTATGCCGTCTTCTGCTTG |
| **1_R2_WT adapter** | 1  SEP3_MI_ WT | R2 | AATGATACGGCGACCACCGAGATCTACACTCTTTCCCTACACGACGCTCTTCCGATCTNNNNNNNNNNNNNNNNNNNNNNNNNCTGAGATCGGAAGAGCACACGTCTGAACTCCAGTCAC*GCGTAT*ATCTCGTATGCCGTCTTCTGCTTG |
| **2_R2_**  **R3A adapter** | 2  SEP3_MI_ R3A | R2 | AATGATACGGCGACCACCGAGATCTACACTCTTTCCCTACACGACGCTCTTCCGATCTNNNNNNNNNNNNNNNNNNNNNNNNNGACTGATCGGAAGAGCACACGTCTGAACTCCAGTCAC*GCGTAT*ATCTCGTATGCCGTCTTCTGCTTG |
| **3_R2_**  **R3K adapter** | 3  SEP3_MI_ R3K | R2 | AATGATACGGCGACCACCGAGATCTACACTCTTTCCCTACACGACGCTCTTCCGATCTNNNNNNNNNNNNNNNNNNNNNNNNNACTGGATCGGAAGAGCACACGTCTGAACTCCAGTCAC*GCGTAT*ATCTCGTATGCCGTCTTCTGCTTG |
| **4_R2_WT**  **adapter** | 4  SEP3_MI_ WT | R2 | AATGATACGGCGACCACCGAGATCTACACTCTTTCCCTACACGACGCTCTTCCGATCTNNNNNNNNNNNNNNNNNNNNNNNNNTGACGATCGGAAGAGCACACGTCTGAACTCCAGTCAC*GCGTAT*ATCTCGTATGCCGTCTTCTGCTTG |
| **5_R2_**  **R3A adapter** | 5  SEP3_MI_ R3A | R2 | AATGATACGGCGACCACCGAGATCTACACTCTTTCCCTACACGACGCTCTTCCGATCTNNNNNNNNNNNNNNNNNNNNNNNNNCGATGATCGGAAGAGCACACGTCTGAACTCCAGTCAC*GCGTAT*ATCTCGTATGCCGTCTTCTGCTTG |
| **6_R2_**  **R3K adapter** | 6  SEP3_MI_ R3K | R2 | AATGATACGGCGACCACCGAGATCTACACTCTTTCCCTACACGACGCTCTTCCGATCTNNNNNNNNNNNNNNNNNNNNNNNNNTACGGATCGGAAGAGCACACGTCTGAACTCCAGTCAC*GCGTAT*ATCTCGTATGCCGTCTTCTGCTTG |
| **3_R3_**  **R3K adapter** | 3  SEP3_MI_ R3K | R3 | AATGATACGGCGACCACCGAGATCTACACTCTTTCCCTACACGACGCTCTTCCGATCTNNNNNNNNNNNNNNNNNNNNNNNNNACTGGATCGGAAGAGCACACGTCTGAACTCCAGTCAC*CATGGA*ATCTCGTATGCCGTCTTCTGCTTG |
| **6_R3_**  **R3K adapter** | 6  SEP3_MI_ R3K | R3 | AATGATACGGCGACCACCGAGATCTACACTCTTTCCCTACACGACGCTCTTCCGATCTNNNNNNNNNNNNNNNNNNNNNNNNNTACGGATCGGAAGAGCACACGTCTGAACTCCAGTCAC*CATGGA*ATCTCGTATGCCGTCTTCTGCTTG |

**Supplementary Table S4.** Sequencing reads obtained for the sequenced 20 SELEX libraries. The unwanted sequencing reads for the positive control are also listed per SELEX cycle. Those sequencing reads were coming from the pool of samples from the respective amplification round.

| **SELEX library name** | **Probe number barcode (4 nt)** | **SELEX cycle barcode (6 nt)** | **Sequencing reads** |
| --- | --- | --- | --- |
| **1_R0** | CTGA | CTACTG | 8,474,742 |
| **2_R0** | GACT | CTACTG | 6,758,001 |
| **3_R0** | ACTG | CTACTG | 8,696,933 |
| **4_R0** | TGAC | CTACTG | 10,677,735 |
| **5_R0** | CGAT | CTACTG | 5,745,049 |
| **6_R0** | TACG | CTACTG | 5,210,936 |
| **1_R1_SEP3_WT** | CTGA | TGCACC | 9,531,429 |
| **2_R1_SEP3_R3A** | GACT | TGCACC | 8,558,593 |
| **3_R1_SEP3_R3K** | ACTG | TGCACC | 10,678,086 |
| **4_R1_SEP3_WT** | TGAC | TGCACC | 7,952,111 |
| **5_R1_SEP3_R3A** | CGAT | TGCACC | 10,433,236 |
| **6_R1_SEP3_R3K** | TACG | TGCACC | 10,232,211 |
| **1_R2_SEP3_WT** | CTGA | GCGTAT | 2,583,876 |
| **2_R2_SEP3_R3A** | GACT | GCGTAT | 4,950,193 |
| **3_R2_SEP3_R3K** | ACTG | GCGTAT | 8,036,453 |
| **4_R2_SEP3_WT** | TGAC | GCGTAT | 3,268,983 |
| **5_R2_SEP3_R3A** | CGAT | GCGTAT | 2,683,050 |
| **6_R2_SEP3_R3K** | TACG | GCGTAT | 5,252,690 |
| **3_R3_SEP3_R3K** | ACTG | CATGGA | 4,885,694 |
| **6_R3_SEP3_R3K** | TACG | CATGGA | 2,095,311 |
| **Positive control_R1** | CATG | TGCACC | 1,091,176 |
| **Positive control_R2** | CATG | GCGTAT | 11,125,777 |
| **Positive control_R3** | CATG | CATGGA | 12,628,887 |
